# Substantial international variation in the cost of blood group and save and crossmatch: A systematic review

**DOI:** 10.1111/bjh.70370

**Published:** 2026-02-24

**Authors:** Gianluca Fabiano, Rafael Pinedo‐Villanueva, Shona Kirtley, Sophie Cole, Artin Manafi‐Khosroshahi, Bilal Qureshi, Isabel Singleton, Biruk Tsegaye, Wei Shao Tung, Florian Tomini, Hayley G. Evans, Mike F. Murphy, Simon J. Stanworth, Paula Dhiman, Antony J. R. Palmer

**Affiliations:** ^1^ Nuffield Department of Orthopaedics, Rheumatology, and Musculoskeletal Sciences University of Oxford Oxford UK; ^2^ Nuffield Department of Orthopaedics, Rheumatology and Musculoskeletal Sciences, Centre for Statistics in Medicine University of Oxford Oxford UK; ^3^ School of Medicine and Biomedical Sciences University of Oxford Oxford UK; ^4^ Medical Sciences Division University of Oxford Oxford UK; ^5^ The Medical School The University of Sheffield Sheffield UK; ^6^ Wolfson Institute of Population Health Queen Mary University of London London UK; ^7^ NIHR Blood and Transplant Research Unit in Data‐Driven Transfusion Practice, Nuffield Division of Clinical Laboratory Sciences, Radcliffe Department of Medicine University of Oxford Oxford UK; ^8^ NHS Blood and Transplant Oxford University Hospitals NHS Foundation Trust and University of Oxford Oxford UK

**Keywords:** cost, crossmatch, group and save, preoperative blood testing, transfusion practice

## Abstract

Blood transfusion is one of the most common interventions in hospitals. Blood tests that support this widespread practice are considered routine, such as ‘group and save’ (also known as ‘type and screen’), yet there has been very limited evaluation of the needs for these tests and the associated costs. The aim of this systematic review is to synthesise international evidence on the costs of group and save and crossmatch tests and their component processes. Seventy‐two studies published between 2012 and 2023 were identified and data were extracted. Most cost estimates were derived from local institutional sources with few using national reference data, and reporting methods varied widely. Median unit costs for group and save tests were £19.8 (interquartile range [IQR] £11.8–£52.5) overall, £13.8 in the United Kingdom and £57.4 in the United States. Crossmatch costs were £9.6 (IQR £5.9–£37.3). Understanding costs for group and save testing will inform hospital teams making decisions about sample requirements for procedures with a low likelihood of transfusion. It also provides benchmarking to assess the impact of implementing electronic systems to improve safety and efficiency of transfusion practice.

## INTRODUCTION

In the United Kingdom, up to 1.6 million units of red blood cells are issued each year,^1^ making it a very common practice for haematology laboratories. While much of the focus of patient blood management has been on reducing the need for blood transfusion to align with evidence‐based recommendations, less research has been devoted to understanding the cost implications of clinical practice and the resources needed for laboratory support.

Laboratory resources are required in hospital blood banks to support all steps of safe matching of blood for patients. Group and save (also known as type and screen) is an essential part of the work‐up of surgical patients to ensure that blood is available for transfusion if it is needed as well as medical patients with anaemia. Group and save is performed on a venous sample to determine the patient's ABO and D blood groups and to screen for red cell antibodies. The blood sample is retained for crossmatching blood for transfusion if it is required.

In haematology practice, patients may require repeated transfusions over months and years. Patients are often expected to attend hospital for multiple group and save tests, often separately from hospital visits for the actual transfusion episode. Current guidance is that patients should have two group and save tests performed before a surgical procedure, using two samples collected on separate occasions, to minimise the risk of error and wrong transfusion.^2^ The elective backlog and waiting times for surgery often increase the need for repeat group and save tests since samples expire after 3 months.

Group and save is recommended before many surgical procedures, even when transfusion rates are low, to ensure matched blood is available if unexpectedly required. The impact of this repeated sampling activity is both to the patient, with repeated hospital visits for venepuncture and contribution to iatrogenic anaemia, and to the hospital with the added cost in materials and staff time both obtaining and processing the samples. Unnecessary testing increases healthcare costs, carbon footprint and is detrimental to patient experience.^3^, ^4^, ^5^, ^6^

Improvements in the safety of sample collection for transfusion using electronic solutions reduce the group and save requirement to one sample.^2^ There is interest from both clinical and patient groups in removing group and save sampling for surgical procedures with a low likelihood of transfusion, with national and international variation in practice. As effective blood conservation strategies become more widespread and the risk of transfusion continues to fall, the requirement for group and save is being revisited by the Getting It Right First Time (GIRFT) programme^7^ in the English National Health Service (NHS), particularly for high‐volume, low‐complexity procedures such as hip and knee replacement. Understanding the cost of group and save testing will inform teams making decisions about indications for group and save and the implementation of electronic systems to improve safety and efficiency.

Currently, the cost of a group and save test is not known, and therefore, it is not possible to identify the potential benefits of modifying selection criteria for a group and save.^8^ Commonly used sources for economic estimations in the United Kingdom such as the Unit Costs of Health and Social Care Programme^9^ and the National Schedule of NHS Costs^10^ do not include reference unit costs for group and save or crossmatch tests, which are essential for estimating potential savings from more targeted testing strategies.

The aim of this systematic review is to synthesise international evidence on the costs of group and save and crossmatch tests and describe the reported activities that contribute to the cost. This review forms an initial stage of a programme of research aimed at understanding potential inefficiencies in common haematology laboratory practices.

## METHODS

We conducted a systematic review of health economic studies reporting the cost of group and save and crossmatch blood testing. The protocol was registered on the international prospective register of systematic reviews (PROSPERO, Evidence for Policy & Practice Information ID: CRD42022366183). The study is reported following the Preferred Reporting Items for Systematic Reviews and Meta‐Analyses (PRISMA) statement and PRISMA‐S (an extension to the PRISMA Statement for Reporting Literature Searches in Systematic Reviews)^11^, ^12^ (Supporting Information B—Table 7).

### Eligibility criteria

Studies reporting unit costs for group and save, crossmatch or any associated test components (e.g. ABO typing, Rh typing, antibody screening/identification) and related processes (e.g. blood collection, analysis) were searched, without restrictions on clinical speciality, country or study design. Our search was limited to studies published between 2012 and 2023 to ensure the data are applicable to current practice. Eligible studies were those published in English excluding abstracts, book chapters and conference abstracts or proceedings.

### Information sources and search strategy

We searched for eligible studies using MEDLINE (via OVID), Web of Science (via Clarivate Analytics), Transfusion Evidence Library (via Evidentia Publishing), Embase (via OVID), INAHTA (https://database.inahta.org/), Cochrane (via Wiley) and CINAHL (via EBSCOHost). The search strategy was run on 17 February 2023 and consisted of a combination of blood test (e.g. ‘group and save’, ‘type and screen’, ‘crossmatch’) and economic (e.g. ‘unit cost’, ‘economic evaluation’) search terms. The full search strategy is reported in Supporting Information A—Table 1.

### Selection process

Studies identified by the search strategy were imported into EndNote citation manager^13^ and deduplicated, then imported into Rayyan^14^ where remaining duplicates were removed and the title and abstract were screened for inclusion. Six independent reviewers (YT, PD, GF, BT, SC, BQ) screened the titles, abstracts and full texts applying the eligibility criteria with reasons for exclusion recorded.

### Data collection and data items

All included papers were allocated to two independent reviewers (GF, AMK, WST or YT) each extracting relevant data independently using a standardised extraction form created in RedCap (Supporting Information A—Table 2). The extraction form was piloted by GF. Disagreements were resolved between the two reviewers and adjudicated by an additional reviewer (RPV). Data items included descriptive characteristics of the study (e.g. country, year of publication, contextual study design), primary outcomes (e.g. unit cost of group and save and crossmatch blood test), secondary outcomes (e.g. cost by test components, activity and costing methods or sources).

### Results synthesis

Data were summarised using descriptive statistics and narrative synthesis. Cost information was synthesised by calculating the mean (standard deviation, SD) and median (interquartile range, IQR) cost per test, per patient and by test components. Costs were further analysed by country and clinical speciality. Currency values were adjusted for inflation and converted to 2022 British pounds using the Evidence for Policy & Practice Information (EPPI) Centre's cost conversion tool^15^ maintained by University College London. Organisation for Economic Co‐operation and Development (OECD) data were used for Purchasing Power Parities (PPP). For studies where OECD PPP values were not available, data from the International Monetary Fund were used. When the price year was not reported, it was assumed to be 1 year earlier than the study publication year. Costs associated with the testing process (i.e. labour and activities) were not synthesised due to variations in how healthcare professional roles and processing activities are defined and reported across studies. All costs in their original currency and year value are presented in Supporting Information B—Tables 1–3. The analysis was conducted using the R programming language.^16^ The quality of the studies was assessed using the Clinical Appraisal Skills Programme (CASP) checklist^17^ for cohort studies, Joanne Briggs Institute (JBI) checklist for case series^18^ and for economic evaluations the Drummond checklist was applied (Supporting Information B—Tables 8–11).^19^

## RESULTS

**FIGURE 1:**
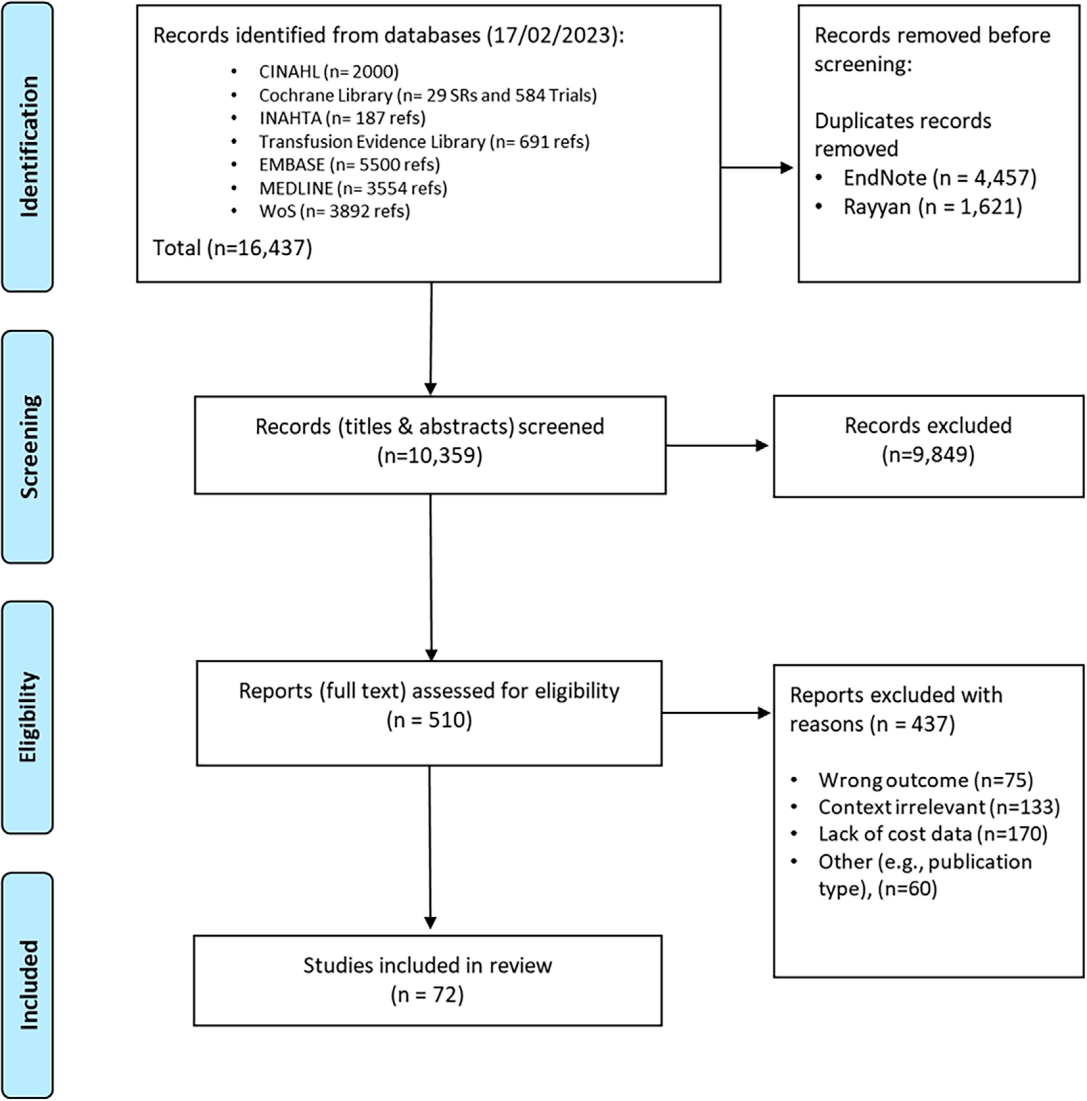
Preferred reporting items for systematic reviews and meta‐analyses (PRISMA) flow diagram.

The search identified 16 437 studies published between 1 January 2012 and 17 February 2023. After screening titles and abstracts, 9849 records were excluded. The full text of 510 studies was assessed for eligibility and 72 studies were included in the review (Figure 1).

### Study characteristics

Of the 72 included studies, most were from the United States (*n* = 30, 41.7%) and the United Kingdom (*n* = 23, 31.9%) (Table 1). Cost information was mostly drawn from clinical observational studies (*n* = 61, 84.7%), which were not specifically designed for economic analysis.

**TABLE 1:** Characteristics of the studies included in the review.

Study characteristics (*n* = 72)	*N* studies	%
Outcomes: preoperative blood tests		
Group and save	37	51.4
Group and save & crossmatch	28	38.9
Crossmatch	7	9.7
Country		
USA	30	41.7
UK	23	31.9
Germany	3	4.2
Canada	2	2.8
Thailand	2	2.8
Other^a^	12	16.7
Contextual study design		
Case series	29	40.3
Chart review	14	19.4
Cohort	9	12.5
Economic evaluation (EE)	8	11.1
Type of EE (*n* = 8)		
Cost‐effectiveness	5	62.5
Cost benefit	2	25.0
Cost analysis	1	12.5
Type of EE model (*n* = 8)		
Decision tree	4	50
Markov	3	37.5
No model	1	12.5
Quasi‐experimental before and after	3	4.2
Micro costing	2	2.8
Systematic review	2	2.8
Case–control	2	2.8
Macro cost analysis	1	1.4
Case note review	1	1.4
Audit	1	1.4
Time approach		
Retrospective	58	80.6
Prospective	6	8.3
Simulation	7	9.7
Cross‐sectional	1	1.4
Clinical speciality		
Surgery	55	76.4
Transfusion medicine	4	5.6
Haematology	3	4.2
Internal medicine	3	4.2
All areas	3	4.2
Obstetrics and gynaecology	2	2.8
Emergency medicine	1	1.4
Oncology	1	1.4
Surgery type (*n* = 55)		
Elective/planned	33	60
All	15	27.3
Emergency/urgent	6	10.9
Not stated	1	1.8
Centres		
Single centre	59	81.9
Multicentre	11	15.3
Not reported	2	2.8

^a^ Studies from ‘other’ countries include one each from Australia, India, Malaysia, Nigeria, Pakistan, Portugal, Saudi Arabia, Singapore, South Korea, Sweden, Turkey and Zimbabwe.

Only 11 studies (15.1%) had a specific economic focus, comprising economic evaluations (*n* = 8), micro‐costing (*n* = 2) or cost analyses (*n* = 1). Of these, six studies examined group and save/type and screen or crossmatch as their primary focus, while the remaining five incorporated these procedures as defined cost components or activities within broader evaluations of transfusion costs, blood product production or disease management. Further details are provided in Supporting Information B—Table 4.

Fifty‐five studies (76.4%) examined surgical interventions and 7 (9.7%) focused on haematology patients. Among the 55 surgical studies, 11 (20%) were for orthopaedic surgery and 8 (14.5%) for general surgery (Supporting Information B—Table 5).

Fifty‐nine studies provided cost information for group and save. Of these, 48 (81.4%) reported the unit cost of the group and save test, 12 (20.3%) detailed the cost of at least one group and save test component (ABO, RhD, antibody screen or antibody identification), 9 (15.3%) provided the cost per patient and 6 (11.2%) provided information on the cost of laboratory services.

Twenty‐seven studies reported cost information for crossmatch. Of these, 23 (85.2%) reported the unit cost of the crossmatch test, and seven (25.9%) provided the cost of at least one crossmatch test component (indirect anti‐globulin test or electronic). In nine studies, costs were reported in an aggregated form (group and save + crossmatch), with two reporting the overall cost per patient.

In 41 studies (56.9%), unit cost was estimated or sourced from the institution performing group and save/crossmatch. In 13 cases (18.1%), cost information originated from cited literature sources. In 10 (13.9%), the source of unit costs was not specified. Eight studies (11.1%) cited reference costs. Forty‐one studies (56.9%) reported cost estimates based on resource use or institutional accounting systems, most commonly from the United Kingdom (*n* = 16) and United States (*n* = 13). Seventeen studies (23.6%) reported fee‐schedule or charge‐based prices, the majority from the United States (*n* = 11). In 14 studies (19.4%), the valuation approach was not reported clearly enough to determine whether values reflected costs or prices.

### Cost results

The median unit cost of group and save and crossmatch when reported in aggregated form was £31.9 (IQR: £12.8–£106.0, *n* = 7). Two studies reported the aggregated cost per patient (median: £44.3, IQR: £40.4–£48.1), as shown in Table 2. All costs in their original currency and year value are presented in Supporting Information B—Tables 1–3. Aggregated mean values are shown in Supporting Information B—Table 6.

**TABLE 2:** Cost results by components.

	N studies	Median (IQR) (£, 2022)
Group and save (*n* = 59)		
Unit cost	48	19.8 (11.8–52.5)
Cost by component		
Blood typing (ABO)	6	22.9 (8.2–64)
RhD typing	4	19.9 (14.3–23.3)
Antibody screen	5	15 (12.4–66.8)
Antibody identification	8	39.7 (33.6–166)
Cost per patient	9	39.1 (29.3–141.1)
Crossmatch (*n* = 27)		
Unit cost	23	9.6 (5.9–37.3)^a^
Cost by component		
Indirect antiglobulin	3	20.5 (10.8–21.7)
Electronic	5	5.5 (3.1–12.4)
Group and save + crossmatch (*n* = 9)		
Unit cost	7	31.9 (12.8–106)
Cost per patient	2	44.3 (40.4–48.1)

Abbreviation: IQR, interquartile range: 25th–75th percentile.

^a^ Unit cost by Mann et al. includes the cost of transfusion and it was excluded from the mean and median calculations.

#### Group and save

**FIGURE 2:**
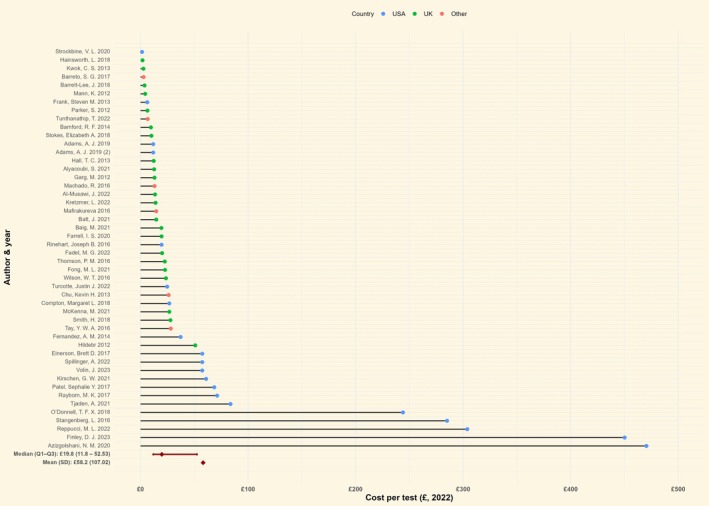
Unit cost of group and save by country (*n* = 48). The figure includes only studies where the unit cost of group and save is reported (*n* = 48). For studies that reported costs of individual group and save components (*n* = 11), these were not aggregated within the study to derive the overall unit cost of group and save and are therefore excluded from this figure. Currency values were adjusted for inflation and converted to British pounds.

The median unit cost of group and save among 48 studies was £19.8 (IQR: £11.8–£52.5) as illustrated in Figure 2 and Table 2. In the 22 UK‐based studies (*n* = 22/48, 45.8%), the median unit cost was £13.8 (IQR: £9.8–£21.9), while in studies from USA (*n* = 20/48, 41.6%), it was £57.4 (IQR: £23.5–£123.8), graphically shown in Supporting Information B—Figure 1.

#### Group and save—Cost components

The median cost of ABO testing across studies (*n* = 6) was £22.9 (IQR: £8.2–£64) as shown in Table 2 and Supporting Information B. In studies from the United States (*n* = 4), the median cost for ABO was £40.5 (IQR: £6.2–£75.6). Only one UK‐based study reported ABO cost of £13.50.

For Rh typing, all studies were from the United States (*n* = 4) with a median cost of £19.9 (IQR: £14.3–£23.3). Additionally, three studies reported a combined median cost of ABO/Rh (£15.0, IQR: £8.8–£20.7).

Similarly, studies reporting separately the costs of antibody screening and identification were mostly from the United States (*n* = 4 and *n* = 10, respectively), with only one study reporting antibody screening costs from Sweden. Overall, the median cost of antibody screening and identification was £15.0 (IQR: £12.4–£66.8) and £39.7 (IQR: £33.6–£166.0) respectively.

Finley et al.^20^ and Azizgolshani, Porter^21^ report the institutional charge combined for serological ABO, Rh type and antibody screen from the Centers for Medicare & Medicaid Services (CMS) fee schedule which was £136.8 (IQR: £135.3–£138.3), with an additional £201.6 (IQR: £199.4–£203.9) for identification in case the screen is positive.

#### Crossmatch

**FIGURE 3:**
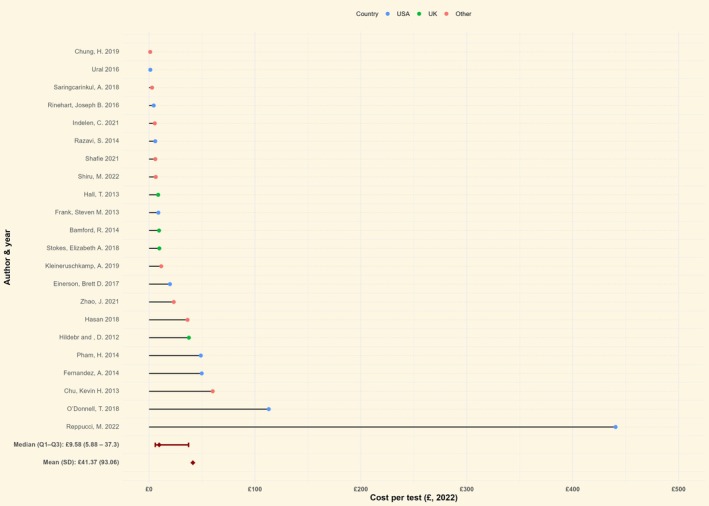
Unit cost of crossmatch by country (*n* = 23). The figure includes only studies where the unit cost of crossmatch is reported (*n* = 23). For studies that reported costs of individual crossmatch components (*n* = 4), these were not aggregated within the study to derive the overall unit cost of group and save and are therefore excluded from this figure. Currency values were adjusted for inflation and converted to British pounds.

The median unit cost of crossmatch test for the 23 studies reporting it was £9.6 (IQR: £5.9–£37.3) (Figure 3; Table 2). Restricting our sample to studies from the United Kingdom (*n* = 4/23, 21.7%) gives a median unit cost of £9.6 (IQR: £9.2–£16.7), whereas for the nine studies from the United States, the median cost was £19.7 (IQR: £5.9–£49.7) (Supporting Information B—Figure 2). Mann et al. (UK) reported the combined cost of crossmatch testing and transfusion (£172.0); therefore, it was excluded from the mean and median calculations (Table 2).

#### Crossmatch—Cost components

Six studies reported on the costs of crossmatch processes, reported in Table 2 and Supporting Information B. Electronic crossmatch costs were reported in five studies: one UK‐based study^22^ estimated the cost based on staff expenses (£3.1), while studies from the USA showed a median cost of £12.4 (IQR £8.9–£14.3).

### Operational time and resource use

#### Time

The total time required to complete group and save ranged from 30 to 120 min.^23^, ^24^, ^25^, ^26^, ^27^, ^28^ A crossmatch was typically performed within 60 min of receiving the group and save result.^23^, ^27^ In cases where patient‐specific crossmatched blood is unexpectedly required after only a group and save test, the crossmatch process took approximately 45–60 min from the time of the request to delivery.^23^, ^27^ Drawing a sample typically takes between 3.1 and 17.8 min.^29^, ^30^, ^31^ Reported durations were derived mainly from institutional estimates of laboratory processing times^23^, ^24^, ^25^, ^26^, ^28^ while only two studies obtained observed process times through direct measurement^29^, ^30^ and one from modelled assumptions.^31^

#### Labour costs, processing activities and consumables

Labour, processing activity and consumable costs varied across studies and are reported as values converted to 2022 British pounds alongside the original study currency and year, reflecting differences in how professional roles, laboratory workflows and procurement practices are defined and utilised across settings.

A US study by Pham, Kim^32^ reported that technologists are paid the equivalent of £20.0 ($25.0) per hour, while in Canada Obaidallah, Downie^29^ provides further details with lab technologists, nurses and porters earning £29.6 (Canadian Dollars, CAD 52.9), £33.5 (CAD 59.9) and £16.1 (CAD 28.8) per hour respectively.

Several studies also examined the cost and time of specific processing activities. In Canada, the cost per activity was estimated at £0.2 (CAD 0.7) for a porter delivering a tube to the blood draw site (average 1.5 min), £1.7 (CAD 3.1) for nursing time to draw a blood group sample (average 3.1 min) and £3.8 (CAD 6.7) for a technologist to process and result a second group sample (average 7.7 min).^29^ In the United States, the nurse cost for drawing a sample was £0.3 ($0.4, 10 min) compared to £0.2 ($ 0.3, 10 min) for that of a phlebotomist. A technologist had the same cost for processing the sample.^31^ A separate US study which measured the full range of activities including accessing pending laboratory orders, collecting supplies, locating the patient room and performing hand hygiene, confirming patient identity and counselling the patient, disinfecting the site and performing the phlebotomy, labelling tubes at the bedside and disposing of materials with final hand hygiene, reported a total time of 17 min, equivalent to £6.5 ($8.6) in phlebotomist staffing costs or £13.8 ($18.4) for a registered nurse.^33^ A UK study by Stokes, Wordsworth^30^ reported a staff time cost of £6.4 for a blood sample and request blood.

Several studies reported higher laboratory costs associated with antibody presence or serological complexity, with those examining serological workups showing approximately twofold to threefold higher costs in antibody‐positive or complex cases.^32^, ^34^, ^35^ Other studies supported the same pattern qualitatively, indicating that antibody presence increases technologist time, testing steps and the need for phenotype‐matched units.^36^, ^37^, ^38^ The cost of consumables also varied. In the United States, Compton, Szklarski^36^ reported a total cost of £1.3 ($1.7) for phlebotomy supplies (including items such as needles, tubes, tourniquets, gloves and biohazard bags) and £6.7 ($9.3) for reagents per test. Bawazir and Dakkam^39^ (USA) reported the cost of reagent identification gel cards for crossmatch at £0.98 ($1.35). In the United Kingdom, Stokes, Wordsworth^30^ lists consumables at £2.8 per test.

Barrett‐Lee, Vatish^40^ found that additional labour and disposable costs for a second sample total £19.7. Chung, Hur^36^ compared automated and manual blood collection management systems, finding that the manual system incurs higher labour costs but lower consumable costs compared to the automated system.

## DISCUSSION

There is considerable variation in the reported unit costs of group and save blood tests and their components across healthcare systems internationally.

Differences were highlighted both by country and by valuation approach. US studies reported higher values than those from the United Kingdom, with median group and save costs around four times higher and crossmatch costs roughly twice as high. Price or fee‐schedule values were also greater than resource‐based cost estimates, with both group and save and crossmatch showing approximately 1.7‐fold differences. Five studies from the USA^20^, ^21^, ^41^, ^42^, ^43^ reported unit costs for group and save and, in two studies, also for crossmatch, that were unexpectedly higher than in other studies, exceeding the 90th percentile. These US figures reflect the use of institutional charges or fee schedule rates, the amounts providers are permitted to bill insurers such as Medicare or private payers, rather than actual costs of service delivery. In contrast, other US‐based studies^31^, ^44^, ^45^ reported costs derived from internal accounting systems based on actual resource use, with substantially lower values. Two studies by Adams et al.^46^, ^47^ used pricing from the Healthcare Blue Book, an online price‐comparison tool reporting ‘fair prices’ based on the actual amounts paid on claims, rather than the amounts billed by providers.^48^

In the United Kingdom, test costs showed less variability, with mean reported unit costs significantly lower than those in the United States. However, reference unit cost remains unavailable, and most UK‐based studies either used internal costing methods (*n* = 15, 65%) or did not disclose the source of cost data (*n* = 5, 22%), limiting comparability. Beyond simple differences in country and valuation approach, the evidence base was heterogeneous in several important respects. Studies varied in clinical setting, valuation method (e.g. cost versus price/fee) and components included. Reporting formats also differed, with some studies providing unit test costs and others only aggregated per patient or bundled laboratory charges. Together with differences in wage structures, laboratory automation and crossmatch technology, these factors explain part of the between‐study spread and limit the extent to which a single pooled value can be treated as a universal benchmark.

### Context and implications

The incidence of blood transfusions is low following procedures such as laparoscopic cholecystectomy and appendicectomy, hernia repairs, breast cancer surgeries,^24^, ^26^, ^40^, ^49^, ^50^, ^51^ and hip and knee arthroplasty.^8^ Among the studies with a specific economic focus on group and save or crossmatch,^32^, ^34^, ^38^, ^52^, ^53^, ^54^ routine preoperative testing was not cost‐effective in low‐risk surgical contexts. Selective testing strategies, such as extending sample validity to reduce repeat tests,^52^ or targeted crossmatching for high‐risk patients^32^ consistently demonstrated potential for substantial cost savings without compromising safety. Therefore, the current routine use of group and save/crossmatch in low‐risk procedures might contribute to unnecessary healthcare spending, iatrogenic anaemia and detriment to patient experience.

This review aimed to provide cost data that can inform the outcomes of more selective testing strategies. In the absence of established reference unit costs for group and save and crossmatch, our synthesis offers a valuable resource for care providers and commissioners seeking to model the potential savings from targeted testing approaches. However, given this heterogeneity, the figures presented here should be interpreted as indicative ranges. For local budget impact or cost‐effectiveness models, users should treat our estimates as starting values, supplement them with local data where possible and explore wide sensitivity ranges around key parameters.

A commonly cited approach involves selective testing based on individual risk factors, such as cardiovascular disease, anaemia, a history of haematological malignancy or previous transfusion events.^25^, ^39^, ^40^, ^43^, ^49^, ^50^, ^53^, ^55^, ^56^ Other studies recommended implementing a maximum surgical blood ordering schedule, which uses historical data to guide group and save and crossmatch test ordering by procedure type, helping reduce routine test ordering.^45^, ^57^, ^58^, ^59^ Clinical decision support systems integrated into electronic health records can provide real‐time information such as patient blood type or prior group and save results, helping prevent duplicate tests and improve adherence to institutional guidelines.^31^, ^33^

Educational training and procedural adjustments can also reduce unnecessary blood test orders. Adams, Baldwin^46^ found that a significant proportion of unnecessary preoperative lab tests were ordered by junior staff in the emergency department, suggesting that targeted educational initiatives, such as weekly didactic sessions, peer comparisons and cost‐awareness campaigns, could reduce these unnecessary orders. Chu, Wagholikar^60^ implemented a pathology request form that limited junior doctors' ability to order tests without senior consultation, resulting in a 19% decrease in unnecessary test ordering. Similarly, Finley et al.^20^ demonstrated the effectiveness of a quality improvement intervention that combined educational training on group and save utilisation with modifications in the electronic ordering system, achieving an 80% reduction in blood test orders without adverse impacts on patient outcomes.

While efforts to reduce routine testing are increasingly supported by evidence, the balance between cost savings and patient safety must be carefully maintained. Even in low‐risk procedures, clinicians must be prepared for unexpected bleeding events. In many institutions, advances in blood bank logistics now allow for remote or on‐demand blood issue without crossmatch, provided patients have no antibodies or special requirements (e.g. irradiated blood).^61^, ^62^ Such electronic transfusion systems can offer both clinical flexibility and cost‐efficiency.

### Strengths and limitations

To our knowledge, we present the first systematic review to examine the cost of group and save and crossmatch tests. It provides a comprehensive international evaluation of unit costs and identifies the main activities that contribute to these costs, offering a reference point to support the development of standardised reporting of cost.

The study has limitations. Although the CASP checklist was used for quality assessment, it is intended primarily for teaching purposes and does not offer a formal scoring framework. Most included studies received negative or uncertain responses (‘No’ or ‘Can't tell’) to key questions regarding confounding. Similarly, when evaluated using Drummond's economic checklist, many studies did not account for differential timing in costs and outcomes (see Supporting Information B—Tables 6 and 7).

Most included studies were observational and did not adhere to the methodological standards of formal economic evaluations. For example, 10 studies (14%) did not report the source of cost data, while 41 studies (57%) relied on internal cost estimates from the participating hospital or centre. Only a minority of studies cited published sources (*n* = 13, 18%) or used national reference costs (*n* = 8, 11%). Where available, these methodological details are included in the ‘Notes’ column of Tables 1–3 in Supporting Information B.

Few studies provided a detailed breakdown of the group and save or crossmatch cost components. Where such information was reported, it was presented as separate component estimates rather than as a decomposition of total unit costs. Costs related to staff time and processing activities could not be synthesised due to inconsistent reporting across studies and differences in how healthcare roles and workflows were defined. Although this decision avoided producing misleading or non‐comparable estimates, it may have led to underestimation in studies that reported only partial cost components. Only a few studies (*n* = 5) reported costs separately for electronic crossmatch methods and provided limited methodological detail to distinguish these from manual crossmatch procedures. Despite evidence that antibody presence and complexity are important drivers of laboratory workload and cost, most studies analysed this factor as a simple binary variable, limiting assessment of how differing antibody patterns or crossmatch complexity influence total testing costs. Studies evaluated transfusion laboratory processes in different clinical contexts, including preoperative surgical pathways, haematology and chronic transfusion pathways, which may limit the direct transferability of their estimates.

Our findings rely on data reported in individual studies and may be influenced by inconsistencies in costing approaches. In some cases, reported median values for specific cost components exceeded the total cost for the full test, likely due to reporting artefacts. These inconsistencies, combined with differences in health system funding and costing methodologies, may limit the generalisability of our findings and reinforce the need for context‐specific adaptation and sensitivity analysis when applying these data.

### Future research

Future research is needed to provide a more accurate cost estimate of the cost of group and save and crossmatch in different settings. A national audit of current transfusion practice would identify variation in current practice and areas of potential cost savings^63^ and the impact to patients. Making test cost data more accessible and standardised allows NHS trusts to benchmark and optimise their use of preoperative blood tests and inform quality improvement initiatives such as GIRFT to refine patient blood management practices.

## CONCLUSIONS

This study provides an international overview of the costs of group and save and crossmatch tests, revealing substantial variation both between and within countries, with higher costs reported in US‐based studies. In the United Kingdom, this variability reflects the absence of standardised reference for unit costs. Although group and save and crossmatch tests are clinically important for ensuring transfusion safety, emerging evidence suggests that routine preoperative testing may be unnecessary in many surgical contexts where the risk of transfusion is low, with large cost‐savings for healthcare providers and improvement in patient experience.

## AUTHOR CONTRIBUTIONS

**Gianluca Fabiano:** investigation; methodology; validation; visualization; data curation; writing – review and editing; writing – original draft; project administration; formal analysis. **Rafael Pinedo‐Villanueva:** Conceptualization; writing – review and editing. **Shona Kirtley:** Data curation. **Sophie Cole:** Investigation. **Artin Manafi‐Khosroshahi:** Investigation. **Bilal Qureshi:** Investigation. **Isabel Singleton:** Investigation. **Biruk Tsegaye:** Investigation. **Wei Shao Tung:** Investigation. **Florian Tomini:** Conceptualization; writing – review and editing. **Hayley G. Evans:** Writing – review and editing; project administration. **Mike F. Murphy:** Writing – review and editing; conceptualization. **Simon J. Stanworth:** Writing – review and editing; conceptualization; funding acquisition. **Paula Dhiman:** Methodology; conceptualization; writing – review and editing; writing – original draft; investigation; supervision. **Antony J. R. Palmer:** Conceptualization; methodology; writing – review and editing; writing – original draft; funding acquisition.

## FUNDING INFORMATION

The National Institute for Health and Care Research (NIHR) Blood and Transplant Research Unit in Data Driven Transfusion Practice (NIHR203334). The views expressed are those of the author(s) and not necessarily those of the NIHR or the Department of Health and Social Care.

## CONFLICT OF INTEREST STATEMENT

The authors declare no conflicts of interest.

## Supporting information


Data S1.

## Data Availability

Data are available upon request.
