# Supplementary material for: Substantial international variation in the cost of blood group and save and crossmatch: A systematic review
Source: Br J Haematol. 2026 Feb 24;208(4):1196–206. doi: 10.1111/bjh.70370 (PMC13071482; doi:10.1111/bjh.70370)
Supplement: Supplementary file 1 — Data S1. [file BJH-208-1196-s001.docx]

## Supplementary material A – Materials and methods

**Table 1: Full search strategy**

| **CINAHL (via EBSCOHost)**  Search filter: the economic facet of this search strategy has been informed by the NHS EED Economics filter and the CADTH Economic search filter. | |
| --- | --- |
| 1 | TI ("group and save*" or "group & save*" or "group-and-save*" or "group and screen*" or "group typing" or "group and hold" or "screen and hold") OR AB ("group and save*" or "group & save*" or "group-and-save*" or "group and screen*" or "group typing" or "group and hold" or "screen and hold") |
| 2 | TI ("typ* and screen*" or "type-and-screen" or "type-and-screening" or "typ* and antibody screen*" or "type and hold" or "serum hold") OR AB ("typ* and screen*" or "type-and-screen" or "type-and-screening" or "typ* and antibody screen*" or "type and hold" or "serum hold") |
| 3 | TI ("type and cross" or "typing and crossmatching" or "cross-match*" or crossmatch* or "cross match*" or "Cross-Match-to-Transfusion Ratio*" or "crossmatched-to-transfused ratio*" or "transfusion-to-cross-match" or "cross-testing") OR AB ("type and cross" or "typing and crossmatching" or "cross-match*" or crossmatch* or "cross match*" or "Cross-Match-to-Transfusion Ratio*" or "crossmatched-to-transfused ratio*" or "transfusion-to-cross-match" or "cross-testing") |
| 4 | TI ("antiglobulin crossmatch*" or "coagulation test*" or "hold clot" or "clot to hold" or "bb hold" or "hold tube" or "electronic remote blood issue" or "electronic cross-match" or "remote blood issue" or "ERBI" or "MSBOS" or "antibody screen*") OR AB ("antiglobulin crossmatch*" or "coagulation test*" or "hold clot" or "clot to hold" or "bb hold" or "hold tube" or "electronic remote blood issue" or "electronic cross-match" or "remote blood issue" or "ERBI" or "MSBOS" or "antibody screen*") |
| 5 | TI (blood N2 (typ* or group* or order* or management or administ* or component* or requisition* or overordering or "over-ordering" or wastage or bank or utilisation or utilization or reservation* or product* or schedule)) OR AB (blood N2 (typ* or group* or order* or management or administ* or component* or requisition* or overordering or "over-ordering" or wastage or bank or utilisation or utilization or reservation* or product* or schedule)) |
| 6 | TI ("blood support" or "ABO typing" or "ABO/Rh typ*" or "blood supply chain" or "bloods" or "PBM program*" or "PBM strateg*") OR AB ("blood support" or "ABO typing" or "ABO/Rh typ*" or "blood supply chain" or "bloods" or "PBM program*" or "PBM strateg*") |
| 7 | (MH "Blood Grouping and Crossmatching") OR (MH "Blood Group Incompatibility+") OR (MH "Blood Transfusion+") |
| 8 | S1 OR S2 OR S3 OR S4 OR S5 OR S6 OR S7 |
| 9 | TI (economic* or cost or costs or costly or costing or microcosting or budget* or save* or saving* or expensive or expense* or expenditure* or price* or pricing or financ* or fee or fees) OR AB (economic* or cost or costs or costly or costing or microcosting or budget* or save* or saving* or expensive or expense* or expenditure* or price* or pricing or financ* or fee or fees) |
| 10 | TI ("value for money" or "cost-effective*" or "micro-costing") OR AB ("value for money" or "cost-effective*" or "micro-costing") |
| 11 | TI ((resource* or service*) N1 (utilisation or utilization)) OR AB ((resource* or service*) N1 (utilisation or utilization)) |
| 12 | (MH "Economics") OR (MH "Costs and Cost Analysis+") OR (MH "Economic Aspects of Illness") OR (MH "Resource Allocation+") OR (MH "Economic Value of Life") OR (MH "Economics, Pharmaceutical") OR (MH "Fees and Charges+") OR (MH "Budgets") OR (MH "Decision Trees") OR (MH "Health Resource Utilization") OR (MH "Health Resource Allocation") OR (MH "Cost Benefit Analysis") OR (MH "Cost Savings") OR (MH "Cost Control") OR (MH "Health Care Costs") OR (MH "Health Facility Costs") |
| 13 | S9 OR S10 OR S11 OR S12 |
| 14 | S8 and S13 |
| 15 | PY 2012 OR PY 2013 OR PY 2014 OR PY 2015 OR PY 2016 OR PY 2017 OR PY 2018 OR PY 2019 OR PY 2020 OR PY 2021 OR PY 2022 OR PY 2023 |
| 16 | S14 AND S15 |
| **Cochrane Library (via Wiley)**  Search filter: the economic facet of this search strategy has been informed by the NHS EED Economics filter, the SIGN Economic Studies search filter and the CADTH Economic search filter. | |
| 1 | ("group and save*" or "group & save*" or "group-and-save*" or "group and screen*" or "group typing" or "group and hold" or "screen and hold"):ti,ab,kw |
| 2 | ("typ* and screen*" or "type-and-screen" or "type-and-screening" or "typ* and antibody screen*" or "type and hold" or "serum hold"):ti,ab,kw |
| 3 | ("type and cross" or "typing and crossmatching" or "cross-match*" or crossmatch* or "cross match*" or "Cross-Match-to-Transfusion Ratio*" or "crossmatched-to-transfused ratio*" or "transfusion-to-cross-match" or "cross-testing"):ti,ab,kw |
| 4 | ("antiglobulin crossmatch*" or "coagulation test*" or "hold clot" or "clot to hold" or "bb hold" or "hold tube" or "electronic remote blood issue" or "electronic cross-match" or "remote blood issue" or "ERBI" or "MSBOS" or "antibody screen*"):ti,ab,kw |
| 5 | (blood next/2 (typ* or group* or order* or management or administ* or component* or requisition* or overordering or "over-ordering" or wastage or bank or utilisation or utilization or reservation* or product* or schedule)):ti,ab,kw |
| 6 | ("blood support" or "ABO typing" or "ABO/Rh typ*" or "blood supply chain" or bloods or "PBM program*" or "PBM strateg*"):ti,ab,kw |
| 7 | MeSH descriptor: ["Blood Grouping and Crossmatching"] this term only |
| 8 | MeSH descriptor: [Blood Group Incompatibility] explode all trees |
| 9 | MeSH descriptor: [Blood Group Antigens] explode all trees |
| 10 | MeSH descriptor: [Blood Safety] this term only |
| 11 | #1 or #2 or #3 or #4 or #5 or #6 or #7 or #8 or #9 or #10 |
| 12 | (economic* or cost or costs or costly or costing or microcosting or budget* or save* or saving* or expensive or expense* or expenditure* or price* or pricing or financ* or fee or fees):ti,ab,kw |
| 13 | ("value for money" or "cost-effective$" or "micro-costing"):ti,ab,kw |
| 14 | ((resource* or service*) next/1 (utilisation or utilization)):ti,ab,kw |
| 15 | MeSH descriptor: [Costs and Cost Analysis] explode all trees |
| 16 | MeSH descriptor: [Cost-Benefit Analysis] this term only |
| 17 | MeSH descriptor: [Cost Savings] this term only |
| 18 | MeSH descriptor: [Cost Control] explode all trees |
| 19 | MeSH descriptor: [Cost Allocation] this term only |
| 20 | MeSH descriptor: [Cost of Illness] this term only |
| 21 | MeSH descriptor: [Cost Sharing] this term only |
| 22 | MeSH descriptor: [Health Care Costs] this term only |
| 23 | MeSH descriptor: [Hospital Costs] this term only |
| 24 | MeSH descriptor: [Health Expenditures] this term only |
| 25 | MeSH descriptor: [Medical Audit] this term only |
| 26 | MeSH descriptor: [Fees and Charges] explode all trees |
| 27 | MeSH descriptor: [Budgets] explode all trees |
| 28 | MeSH descriptor: [Direct Service Costs] this term only |
| 29 | MeSH descriptor: [Health Resources] this term only |
| 30 | MeSH descriptor: [Facilities and Services Utilization] this term only |
| 31 | MeSH descriptor: [Employer health costs] this term only |
| 32 | MeSH descriptor: [Economics] this term only |
| 33 | MeSH descriptor: [Economics, Hospital] explode all trees |
| 34 | MeSH descriptor: [Economics, Medical] explode all trees |
| 35 | MeSH descriptor: [Economics, Nursing] this term only |
| 36 | MeSH descriptor: [Economics, Pharmaceutical] this term only |
| 37 | #12 or #13 or #14 or #15 or #16 or #17 or #18 or #19 or #20 or #21 or #22 or #23 or #24 or #25 or #26 or #27 or #28 or #29 or #30 or #31 or #32 or #33 or #34 or #35 or #36 |
| 38 | #11 and #37 with Cochrane Library publication date from Jan 2012 to Feb 2023 |
| **EMBASE**  Search filter: the economic facet of this search strategy has been informed by the NHS EED Economics filter, the SIGN Economic Studies search filter and the CADTH Economic search filter. | |
| 1 | ("group and save$" or "group & save$" or "group-and-save$" or "group and screen$" or "group typing" or "group and hold" or "screen and hold").ti,ab,kw. |
| 2 | ("typ$ and screen$" or "type-and-screen" or "type-and-screening" or "typ$ and antibody screen$" or "type and hold" or "serum hold").ti,ab,kw. |
| 3 | ("type and cross" or "typing and crossmatching" or "cross-match$" or crossmatch$ or "cross match$" or "Cross-Match-to-Transfusion Ratio$" or "crossmatched-to-transfused ratio$" or "transfusion-to-cross-match" or "cross-testing").ti,ab,kw. |
| 4 | ("antiglobulin crossmatch$" or "coagulation test$" or "hold clot" or "clot to hold" or "bb hold" or "hold tube" or "electronic remote blood issue" or "electronic cross-match" or "remote blood issue" or "ERBI" or "MSBOS" or "antibody screen$").ti,ab,kw. |
| 5 | (blood adj2 (typ$ or group$ or order$ or management or administ$ or component$ or requisition$ or overordering or "over-ordering" or wastage or bank or utilisation or utilization or reservation$ or product$ or schedule)).ti,ab,kw. |
| 6 | ("blood support" or "ABO typing" or "ABO/Rh typ$" or "blood supply chain" or bloods or "PBM program$" or "PBM strateg$").ti,ab,kw. |
| 7 | Blood group typing/ or exp Blood group incompatibility/ or exp Blood group antigen/ or Blood safety/ or exp Blood transfusion/ |
| 8 | or/1-7 |
| 9 | (economic$ or cost or costs or costly or costing or microcosting or budget$ or save$ or saving$ or expensive or expense$ or expenditure$ or price$ or pricing or financ$ or fee or fees).ti,kf. |
| 10 | (economic$ or cost or costs or costly or costing or microcosting or budget$ or save$ or saving$ or expensive or expense$ or expenditure$ or price$ or pricing or financ$ or fee or fees).ab. /freq=2 |
| 11 | ("value for money" or "cost-effective$" or "micro-costing").ti,ab,kw. |
| 12 | ((resource$ or service$) adj1 (utilisation or utilization)).ti,ab,kw. |
| 13 | Cost benefit analysis/ or Cost effectiveness analysis/ or Cost utility analysis/ or Cost of illness/ or Cost control/ or Cost minimization analysis/ or Economic evaluation/ |
| 14 | Health care cost/ or Health care financing/ or Hospital cost/ or Cost/ or Budget/ |
| 15 | Health economics/ or Economics/ or Microcosting/ |
| 16 | or/9-15 |
| 17 | 8 and 16 |
| 18 | conference abstract.pt. |
| 19 | conference abstract.st. |
| 20 | 18 or 19 |
| 21 | 17 not 20 |
| 22 | limit 21 to yr="2012-2023" |
| **INAHTA International HTA Database via https://database.inahta.org/**  Search filter: the economic facet of this search strategy has been informed by the NHS EED Economics filter, the SIGN Economic Studies search filter and the CADTH Economic search filter. | |
| 1 | "group and save*" or "group & save*" or "group-and-save*" or "group and screen*" or "group typing" or "group and hold" or "screen and hold" in ALL |
| 2 | "typ* and screen*" or "type-and-screen" or "type-and-screening" or "typ* and antibody screen*" or "type and hold" or "serum hold" in ALL |
| 3 | "type and cross" or "typing and crossmatching" or "cross-match*" or crossmatch* or "cross match*" or "Cross-Match-to-Transfusion Ratio*" or "crossmatched-to-transfused ratio*" or "transfusion-to-cross-match" or "cross-testing" in ALL |
| 4 | "antiglobulin crossmatch*" or "coagulation test*" or "hold clot" or "clot to hold" or "bb hold" or "hold tube" or "electronic remote blood issue" or "electronic cross-match" or "remote blood issue" or "ERBI" or "MSBOS" or "antibody screen*" in ALL |
| 5 | blood* in ALL |
| 6 | "Blood Group Incompatibility"[mhe] or "Blood Grouping and Crossmatching"[mh] or "Blood Group Antigens"[mhe] or "Blood Safety"[mh] |
| 7 | #6 OR #5 OR #4 OR #3 OR #2 OR #1 |
| 8 | economic* or cost or costs or costly or costing or microcosting or budget* or save* or saving* or expensive or expense* or expenditure* or price* or pricing or financ* or fee or fees in ALL |
| 9 | "value for money" or "cost-effective*" or "micro-costing" or "resource* utilisation" or "service* utilisation" or "resource* utilization" or "service* utilization" in ALL |
| 10 | "Costs and Cost Analysis"[mhe] or "Cost-Benefit Analysis"[mh] or "Cost Savings"[mh] or "Cost Control"[mh] or "Cost Allocation"[mh] or "Cost of Illness"[mh] or "Cost Sharing"[mh] |
| 11 | "Health Care Costs"[mh] or "Hospital Costs"[mh] or "Health Expenditures"[mh] or "Medical Audit"[mh] or "Fees and Charges"[mhe] or Budgets[mhe] or "Direct Service Costs"[mh] or "Health Resources"[mh] or "Facilities and Services Utilization"[mh] |
| 12 | "Employer health costs"[mh] or Economics[mh] or "Economics, Hospital"[mhe] or "Economics, Medical"[mhe] or "Economics, Nursing"[mh] or "Economics, Pharmaceutical"[mh] |
| 13 | #12 OR #11 OR #10 OR #9 OR #8 |
| 14 | #13 AND #7 |
| 15 | Limit to year "2012-2023" |
| **Medline** (Ovid MEDLINE® Epub Ahead of Print, In-Process & Other Non-Indexed Citations, Ovid MEDLINE® Daily and Ovid MEDLINE®) 1946 to present. Searched via OVID.  Search filter: the economic facet of the this search strategy has been informed by the NHS EED Economics filter, the SIGN Economic Studies search filter and the CADTH Economic search filter. | |
| 1 | ("group and save$" or "group & save$" or "group-and-save$" or "group and screen$" or "group typing" or "group and hold" or "screen and hold").ti,ab,kw. |
| 2 | ("typ$ and screen$" or "type-and-screen" or "type-and-screening" or "typ$ and antibody screen$" or "type and hold" or "serum hold").ti,ab,kw. |
| 3 | ("type and cross" or "typing and crossmatching" or "cross-match$" or crossmatch$ or "cross match$" or "Cross-Match-to-Transfusion Ratio$" or "crossmatched-to-transfused ratio$" or "transfusion-to-cross-match" or "cross-testing").ti,ab,kw. |
| 4 | ("antiglobulin crossmatch$" or "coagulation test$" or "hold clot" or "clot to hold" or "bb hold" or "hold tube" or "electronic remote blood issue" or "electronic cross-match" or "remote blood issue" or "ERBI" or "MSBOS" or "antibody screen$").ti,ab,kw. |
| 5 | (blood adj2 (typ$ or group$ or order$ or management or administ$ or component$ or requisition$ or overordering or "over-ordering" or wastage or bank or utilisation or utilization or reservation$ or product$ or schedule)).ti,ab,kw. |
| 6 | ("blood support" or "ABO typing" or "ABO/Rh typ$" or "blood supply chain" or bloods or "PBM program$" or "PBM strateg$").ti,ab,kw. |
| 7 | "Blood Grouping and Crossmatching"/ or exp Blood Group Incompatibility/ or exp Blood Group Antigens/ or Blood Safety/ |
| 8 | or/1-7 |
| 9 | (economic$ or cost or costs or costly or costing or microcosting or budget$ or save$ or saving$ or expensive or expense$ or expenditure$ or price$ or pricing or financ$ or fee or fees).ti,ab,kf. |
| 10 | ("value for money" or "cost-effective$" or "micro-costing").ti,ab,kw. |
| 11 | ((resource$ or service$) adj1 (utilisation or utilization)).ti,ab,kw. |
| 12 | exp "Costs and Cost Analysis"/ or Cost-Benefit Analysis/ or Cost Savings/ or Cost Control/ or Cost Allocation/ or Cost of Illness/ or Cost Sharing/ |
| 13 | Health Care Costs/ or Hospital Costs/ or Health Expenditures/ or Medical Audit/ or exp "Fees and Charges"/ or exp Budgets/ or Direct Service Costs/ or Health Resources/ or "Facilities and Services Utilization"/ |
| 14 | Employer health costs/ or Economics/ or exp Economics, Hospital/ or exp Economics, Medical/ or Economics, Nursing/ or Economics, Pharmaceutical/ |
| 15 | or/9-14 |
| 16 | 8 and 15 |
| **Transfusion Evidence Library (via http://www.transfusionevidencelibrary.com/)**  Search filter: the economic facet of the this search strategy has been informed by the NHS EED Economics filter, the SIGN Economic Studies search filter and the CADTH Economic search filter. | |
| 1 | economic* OR cost OR costs OR costly OR costing OR microcosting OR budget* OR save* OR saving* OR expensive OR expense* OR expenditure* OR price* OR pricing OR financ* OR fee OR fees OR "value for money" OR "cost-effective*" OR "micro-costing" (in main search box on homepage, no fields specified) |
| **Web of Science Core Collection** (via Clarivate Analytics at https://www.webofscience.com/wos/woscc/)  Editions searched: Science Citation Index Expanded (SCI-EXPANDED), Conference Proceedings Citation Index – Science (CPCI-S).  Search filter: the economic facet of the this search strategy has been informed by the NHS EED Economics filter, the SIGN Economic Studies search filter and the CADTH Economic search filter. | |
| 1 | TS=("group and save*" OR "group & save*" OR "group-and-save*" OR "group and screen*" OR "group typing" OR "group and hold" OR "screen and hold") |
| 2 | TS=("typ* and screen*" OR "type-and-screen" OR "type-and-screening" OR "typ* and antibody screen*" OR "type and hold" OR "serum hold") |
| 3 | TS=("type and cross" OR "typing and crossmatching" OR "cross-match*" OR crossmatch* or "cross match*" OR "Cross-Match-to-Transfusion Ratio*" OR "crossmatched-to-transfused ratio*" OR "transfusion-to-cross-match" OR "cross-testing") |
| 4 | TS=("antiglobulin crossmatch*" OR "coagulation test*" OR "hold clot" OR "clot to hold" OR "bb hold" OR "hold tube" OR "electronic remote blood issue" OR "electronic cross-match" OR "remote blood issue" OR "ERBI" OR "MSBOS" OR "antibody screen*") |
| 5 | TS=(blood NEAR/2 (typ* OR group* OR order* OR management OR administ* OR component* OR requisition* OR overordering OR "over-ordering" OR wastage OR bank OR utilisation OR utilization OR reservation* OR product* OR schedule)) |
| 6 | TS=("blood support" OR "ABO typing" OR "ABO/Rh typ*" OR "blood supply chain" OR "bloods" OR "PBM program*" OR "PBM strateg*") |
| 7 | #1 OR #2 OR #3 OR #4 OR #5 OR #6 |
| 8 | TS=(economic* OR cost OR costs OR costly OR costing OR microcosting OR budget* OR save* OR saving* OR expensive OR expense* OR expenditure* OR price* OR pricing OR financ* OR fee OR fees OR "value for money" OR "cost-effective*" OR "micro-costing") |
| 9 | TS=((resource* OR service*) NEAR/1 (utilisation or utilization)) |
| 10 | #8 OR #9 |
| 11 | PY=(2012-2023) |
| 12 | #7 AND #10 AND #11 [Indexes=SCI-EXPANDED, CPCI-S] |

**Table 2: Extraction form (RedCap)**

| Record ID |
| --- |
| Survey Identifier |
| Survey Timestamp |
| Rayyan ID |
| Reviewer initials |
| Country What is the geographic location of the study? if not available, please enter the country affiliation of the first author |
| If Other, please specify: |
| AimWhat is the aim of the study? |
| Study design (choice=Retrospective study) |
| Study design (choice=Cross-sectional) |
| Study design (choice=Economic evaluation) |
| Study design (choice=Cost analysis) |
| Study design (choice=Microcosting) |
| Study design (choice=Other study_design_oth |
| Other |
| Time frame (horizon)What are the start and end dates (years) of data collection? |
| Clinical area (main medical area/condition) (choice=Allergy and immunology) |
| Clinical area (main medical area/condition) (choice=Anesthesiology) |
| Clinical area (main medical area/condition) (choice=Dermatology) |
| Clinical area (main medical area/condition) (choice=Diagnostic radiology) |
| Clinical area (main medical area/condition) (choice=Emergency medicine) |
| Clinical area (main medical area/condition) (choice=Family medicine) |
| Clinical area (main medical area/condition) (choice=Internal medicine) |
| Clinical area (main medical area/condition) (choice=Medical genetics) |
| Clinical area (main medical area/condition) (choice=Neurology) |
| Clinical area (main medical area/condition) (choice=Nuclear medicine) |
| Clinical area (main medical area/condition) (choice=Obstetrics and gynecology) |
| Clinical area (main medical area/condition) (choice=Ophthalmology) |
| Clinical area (main medical area/condition) (choice=Orthopaedic) |
| Clinical area (main medical area/condition) (choice=Pathology) |
| Clinical area (main medical area/condition) (choice=Pediatrics) |
| Clinical area (main medical area/condition) (choice=Physical medicine and rehabilitation) |
| Clinical area (main medical area/condition) (choice=Preventive medicine) |
| Clinical area (main medical area/condition) (choice=Psychiatry) |
| Clinical area (main medical area/condition) (choice=Radiation oncology) |
| Clinical area (main medical area/condition) (choice=Surgery) |
| Clinical area (main medical area/condition) (choice=Urology) |
| Clinical area (main medical area/condition) (choice=Other) |
| Other |
| Sample size How many participants were included in the study? |
| Population - age |
| Population - sex (choice=Male) |
| Population - sex (choice=Female) |
| Population - sex (choice=Both) |
| Settings the study conducted in a single or multiple centres? (choice=Single centre) |
| Settings the study conducted in a single or multiple centres? (choice=Multiple centres, please specify number n_hospitals) |
| Number centres |
| Clinical setting (choice=Elective surgery) |
| Clinical setting (choice=Non-surgical) |
| Clinical setting (choice=Emergency/Urgent) |
| Clinical setting (choice=Other clinical_setting_oth) |
| Other clinical setting |
| SurgeryWhat surgery was provided? (Enter name(s) of surgery-ies) |
| Blood sampling process What type of blood sampling process was analysed in the study? (choice=Group and save (also type and screen, or type and cross)) |
| Blood sampling process What type of blood sampling process was analysed in the study? (choice=Crossmatch) |
| Blood sampling process What type of blood sampling process was analysed in the study? (choice=Other blood_sampling_oth) |
| If Other, please specify: |
| Blood test Which pre-operative blood tests were performed? (choice=Blood Group (ABO) - part of group and save) |
| Blood test Which pre-operative blood tests were performed? (choice=RhD typing - part of group and save) |
| Blood test Which pre-operative blood tests were performed? (choice=Antibody screen/detection/identification - crossmatch, typically performed after G&S) |
| Blood test Which pre-operative blood tests were performed? (choice=Other(s) blood_test_oth) |
| Electronic Issue? Electronic Issue is the process whereby blood is issued to a patient without serological crossmatching. |
| Other |
| Main findings |
| Study limitations |
| Currency |
| Other |
| Currency year |
| Sources or methods used for cost information (choice=Literature) |
| Sources or methods used for cost information (choice=Not stated) |
| Sources or methods used for cost information (choice=Internal costing) |
| Sources or methods used for cost information (choice=Reference cost) |
| Sources or methods used for cost information (choice=Tariff) |
| Sources or methods used for cost information (choice=Other sources_costing_oth) |
| Sources for costing |
| Was discounting applied? |
| Group and Save - time |
| Group and Save - total cost |
| Group and save - notes |
| Crossmatch(XM) - add on (not electronic, e.g.:serological) - time |
| Crossmatch(XM) - add on (not electronic, e.g.:serological) - total cost |
| Crossmatch(XM) - add on (not electronic, e.g.:serological) - notes |
| Crossmatch (XM) - add on (electronic issue) - time |
| Crossmatch (XM) - add on (electronic issue) - tot cost |
| Crossmatch (XM) - add on (electronic issue) - notes |
| GS + XM (not electronic) - time |
| GS + XM (not electronic) - tot cost |
| GS + XM (not electronic) - notes |
| GS + XM (electronic) - time |
| GS + XM (electronic) - tot cost |
| GS + XM (electronic) - notes |
| Blood Group (ABO) - time |
| Blood Group (ABO) - tot cost |
| Blood Group (ABO) - notes |
| Antibody screen/detection/identification - Time |
| Antibody screen/detection/identification - tot cost |
| Antibody screen/detection/identification - notes |
| RhD typing - Time |
| RhD typing - tot cost |
| RhD typing - notes |
| Other test - Time |
| Other test - tot cost |
| Other test - notes |
| Sample collection - Time |
| Sample collection - tot cost |
| Sample collection - notes |
| Sample analysis - Time |
| Sample analysis - tot cost |
| Sample analysis - notes |
| Crossmatching (not electronic) - Time |
| Crossmatching (not electronic) - tot cost |
| Storage blood product - Time |
| Crossmatching (not electronic) - notes |
| Crossmatching (electronic) - tot cost |
| Crossmatching (electronic) - time |
| Crossmatching (electronic) - notes |
| Storage blood product - tot cost |
| Storage blood product - notes |
| Transport - Time |
| Transport - tot cost |
| Transport - notes |
| Disposal - Time |
| Disposal - tot cost |
| Disposal - notes |
| Stock control - Time |
| Stock control - tot cost |
| Stock control - notes |
| Audit trail for blood products - Time |
| Audit trail for blood products - tot cost |
| Audit trail for blood products - notes |
| Other activity - tot cost |
| Other activity - time |
| Other activity - notes |
| Gloves |
| Tourniquets |
| Needle |
| Alcohol |
| Plaster |
| Vacutainer |
| Equipment |
| All consumables |
| Other consumables |
| Consumables - notes |
| Staff Which staff cadres were involved in taking and or analysing blood? (choice=Nurse) |
| Staff Which staff cadres were involved in taking and or analysing blood? (choice=Medical doctor) |
| Staff Which staff cadres were involved in taking and or analysing blood? (choice=Student/Intern) |
| Staff Which staff cadres were involved in taking and or analysing blood? (choice=Blood bank manager) |
| Staff Which staff cadres were involved in taking and or analysing blood? (choice=Lab scientist/technician) |
| Staff Which staff cadres were involved in taking and or analysing blood? (choice=Not stated) |
| Staff Which staff cadres were involved in taking and or analysing blood? (choice=Other staff_cadre_oth) |
| Other |
| Other cost results (please report here aggregate costs if available, or any other cost information) |
| Reviewer comments |

## Supplementary material B - Results

**Table 1: Unit cost for Group and Save (GS).**

| **Study** | **Currency (year)** | **Study design** | **Cost per test (GS)** **^+^** | **GS components – cost per test ^+^** | | | | **Cost per patient (GS) ^+^** | **Notes** | **Costing method** |
| --- | --- | --- | --- | --- | --- | --- | --- | --- | --- | --- |
|  |  |  |  | ***Blood Group (ABO)*** | ***RhD typing*** | ***Antibody screen*** | ***Antibody identification*** |  |  |  |
| Adams, Baldwin (1) | USA $ (2019) | Case series | 16.00 |  |  |  |  |  | Range: (8-40) | Reference cost |
| Adams, Cahill (2) | USA $ (2019) | Case series | 16.00 |  |  |  |  |  | Range: (8-40) | Reference cost |
| Al-Musawi, Reece (3) | UK £ (2022) | Case series | 13.50 | 13.50 |  |  |  | 27.00 | Two samples per patient | Internal costing |
| Alyacoubi, Taj (4) | UK £ (2021) | Case series | 12.00 |  |  |  |  |  |  | Not stated |
| Azizgolshani, Porter (5) | USA $ (2020) | Cohort | 646.00 | 192.00 | | | 283.00 |  | Institutional charges (Centers for Medicare & Medicaid Services  (CMS) schedule, same as [50] | Internal costing |
| Baig, Sarma (6) | UK £ (2021) | Cohort | 18.39 |  |  |  |  | 36.78 | Two samples per patient | Not stated |
| Bamford, Hall (7) | UK £ (2014) | Case note review | 7.98 |  |  |  |  |  |  | Internal costing |
| Barreto, Singh (8) | USA $ (2017) | Cohort | 3.70 |  |  |  |  |  |  | Internal costing |
| Barrett-Lee, Vatish (9) | UK £ (2018) | Case series | 3.29 |  |  |  |  |  |  | Internal costing |
| Batt, Chambers (10) | UK £ (2021) | Case series | 13.88 |  |  |  |  | 27.76 |  | Internal costing |
| Bawazir and Dakkam (11) | USA $ (2020) | Case-control |  | 2.65 | |  |  |  | Reverse grouping | Internal costing |
| Christopher, Verhey (12) | USA $ (2021) | Case series |  | 106.97 | 32.89 |  | 51.41 | 191.27 |  | Reference cost |
| Chu, Wagholikar (13) | Australian $ (2012) | Quasi-experimental before-and-after | 43.13 |  |  |  |  |  |  | Reference cost |
| Compton, Szklarski (14) | USA $ (2018) | Case series | 30.73-40.5 |  |  |  |  |  | Cost based on phlebotomist or registered nurse time | Not stated |
| Einerson, Stehlikova (15) | USA $ (2015) | Economic evaluation | 72.54 (31-160)^*^ |  |  |  |  |  |  | Literature |
| Fadel, Patel (16) | UK £ (2021) | Systematic Review | 18.99 (15-21.3)^*^ |  |  |  |  |  | Mean of values from literature | Literature |
| Farrell, Hall (17) | UK £ (2019) | Case series | 17.50 |  |  |  |  | 35.00 | Two samples per patient | Internal costing |
| Fernandez, Cronin (18) | USA $ (2012) | Chart review | 45 |  |  |  |  |  |  | Internal costing |
| Finley, Fay (19) | USA $ (2021) | Cohort | 646.00 | 192.00 | | | 283.00 |  | Institutional charges (Centers for Medicare & Medicaid Services  (CMS) schedule, same as [21] | Internal costing & Reference cost |
| Fong, Rodriguez (20) | UK £ (2018) | Case series | 20.00 |  |  |  |  |  | Two samples per patient | Literature |
| Frank, Rothschild (21) | USA $ (2012) | Case series | 7.56 |  |  |  |  |  | Hospital charge to patients: 37 | Internal costing |
| Garg, Coleman (22) | UK £ (2010) | Chart review | 10.00 |  |  |  |  |  |  | Not stated |
| Parker, Mahawar (23) | UK £ (2011) | Case series | 5.06 |  |  |  |  |  |  | Internal costing |
| Patel, Edwards (24) | USA $ (2016) | Economic evaluation | 87.50 (75-100) ^*^ |  |  |  |  |  | Estimated range given, not specific value. Also, analysis performed based on hospital charge, not actual cost of reagents, disposable items, and personnel time. | Literature |
| Pham, Kim (25) | USA $ (2013) | Economic evaluation |  | 26.46 | | 104.16 | 32.92 |  | Antibody panel interpretation: 50, Technologist time: 50/h | Internal costing |
| Rayborn, Turner (26) | USA $ (2013) | Case series | 75-100^*^ |  |  |  |  |  | Does not include cost of blood bank inventory management. | Literature |
| Reppucci, Meier (27) | USA $ (2021) | Chart review | 436.00 |  |  |  |  |  |  | Internal costing |
| Rinehart, Lee (28) | USA $ (2014) | Case series | 24.50 |  |  |  |  |  | Including laboratory reagents, technician time, and equipment use | Internal costing |
| Saringcarinkul and Chuasuwan (29) | USA $ (2015) | Case series |  |  |  |  |  | 6.52 | Does not cover all the salaries, infrastructure, training, reagents, and supplies for donor blood collection, storage, and testing. | Internal costing |
| Smith, Falconer (30) | UK £ (2016) | Case series | 23.52 |  |  |  |  |  | Two samples per patient. | Internal costing |
| Spillinger, Allen (31) | USA $ (2015) | Economic evaluation | 72.54 (31-160)^*^ |  |  |  |  |  |  | Literature [55] |
| Stangenberg, Curran (32) | USA $ (2013) | Case-control | 350.00 |  |  |  |  |  |  | Internal costing |
| Steele, Herman (33) | USA $ (2019) | Chart review |  |  |  |  | 211 |  |  | Internal costing |
| Stokes, Wordsworth (34) | UK £ (2015) | Microcosting | 8.4 |  |  |  |  |  |  | Internal costing |
| Strockbine, Gehrie (35) | USA $ (2018) | Quasi-experimental before-and-after | 1.86 |  |  |  |  |  | Direct costs only: testing material and reagents | Internal costing |
| Tay, Woo (36) | Singapore dollar (2016) | Chart review | 27.70 |  |  |  |  |  |  | Internal costing |
| Thomson, Ross (37) | UK £ (2013) | Chart review | 18.39 |  |  |  |  |  | Exclude laboratory costs | Internal costing |
| Tjaden, Codispoti (38) | USA $ (2019) | Chart review | 113.39 | 7.39 | 23.00 |  |  | 193.39 |  | Internal costing |
| Tunthanathip, Sae-Heng (39) | USA $ (2022) | Cohort | 10.34 |  |  |  |  |  |  | Not stated |
| Turcotte, Holbert (40) | USA $ (2022) | Case series | 38.00 | 15.00 | |  | 23.00 |  |  | Internal costing |
| Vestermark, Rowe (41) | USA $ (2019) | Case series |  | 106.97 | 32.89 |  | 51.41 | 191.27 | Centers for Medicaid and Medicare Services  (CMS) 2019 payment rates | Reference cost |
| Volin, Daniel (42) | USA $ (2015) | Economic evaluation | 72.54 (31-160)^*^ |  |  |  |  |  | Same reported cost as [55] and [41] | Literature |
| Wilson, Young (43) | UK £ (2016) | Chart review | 20.00 |  |  |  |  |  |  | Not stated |
| Zhao, Dahlen (44) | USA $ (2018) | Case series |  | 43.00 |  | 89.00 |  |  |  | Not stated |
| Hainsworth, Tracy (45) | UK £ (2018) | Case series | 1.65 |  |  |  |  |  |  | Internal costing |
| Hall, Pattenden (46) | UK £ (2013) | Case series | 10.00 |  |  |  |  |  |  | Internal costing |
| Hasan, Khan (47) | USA $ (2018) | Case series |  |  |  |  |  |  |  | Internal costing |
| Hildebrand, Binnie (48) | UK £ (2012) | Case series | 40.6 |  |  |  |  |  | Exclude laboratory staff costs | Internal costing |
| Kacker, Ness (49) | USA $ (2012) | Economic evaluation |  | 7.71 (5.78-9.64) ^*^ | 7.71 (5.78, 9.64) ^*^ | 14.95 (11.21, 18.69) ^*^ | 24.77 (18.58, 30.96) ^*^ |  | Other GS tests: Direct Antiglobulin Test (for patients with positive screen only) $7.71 (5.78, 9.64); Elution (for patients with positive DAT only) $24.77 (18.58, 30.96); Adsorption Study (for patients with positive screen indicating Auto AB only) $24.77 (18.58, 30.96); Negative Antigens (per antigen negative, per unit) $80 (60, 100) | Reference cost |
| Kirschen, Dayton (50) | USA $ (2021) | Cohort | 75-100 |  |  |  |  |  |  | Literature |
| Kretzmer, Damola (51) | UK £ (2022) | Chart review | 14 (4.58-23.52)^*^ |  |  |  |  |  | Value extrapolated as a mean of values from literature | Literature |
| Kwok, White (52) | UK £ (2013) | Audit | 2.20 |  |  |  |  |  |  | Internal costing |
| Machado, Loureiro (53) | Euro (2016) | Chart review | 10.00 |  |  |  |  |  |  | Internal costing |
| Mann, Sim (54) | UK £ (2012) | Case series | 3.48 |  |  |  |  |  |  | Internal costing |
| McKenna and Abdelaal (55) | UK £ (2021) | Chart review | 25.41 |  |  |  |  |  |  | Internal costing |
| O'Donnell, Shean (56) | USA $ (2018) | Cohort | 325.00 |  |  |  |  |  | Medicare reimbursement is $136.36 for a simple type and screen, with an additional $209.49 if any antibodies are positive | Internal costing & Reference cost |
| Mazonson, Efrusy (57) | USA $ (2014) | Case series |  |  |  |  | 7.00 |  |  | Internal costing |
| Mafirakureva, Nyoni (58) | USA $ (2013) | Macro cost analysis | 17.88 |  |  |  |  |  | Included costs of screening for transfusion-transmissible infections and blood grouping,  95% Confidence interval: 17.32-18.42 | Reference cost |
| Obaidallah, Downie (59) | Canadian $ (2022) | Chart review |  |  |  |  |  | 9.83 | Sum of cost for collecting and processing a second blood sample for ABO | Internal costing |

*Legend: ^*^ range (min-max) ^+^ 95% confidence interval, ^+^ cost reported in the original currency value and year. GS: Group and Save*

**Table 2: Unit cost for Crossmatch (CM) test.**

| **Study** | **Currency (year)** | **Study design** | **Cost per test^*^** | **Cost of CM components^*^** | | **Notes** | **Costing method** |
| --- | --- | --- | --- | --- | --- | --- | --- |
|  |  |  |  | **Indirect antiglobulin** | **Electronic** |  |  |
| Bamford, Hall (7) | UK £ (2014) | Case note review | 780 |  |  |  | Internal costing |
| Bawazir and Dakkam (11) | USA $ (2020) | Case-control |  | 1.35 |  | Cost of ID gel cards + reagents of Liss/Coomb  bs (0.98+0.37) | Internal costing |
| Chu, Wagholikar (13) | Australian $ (2012) | Quasi-experimental before-and-after | 99.01 |  |  |  | Reference cost |
| Chung, Hur (60) | USA $ (2018) | Cohort | 1.44 |  | 2.7 |  | Literature |
| Cushing, DeSimone (61) | USA $ (2017) | Cohort |  | 29.8 |  | Cost of reagent and labour | Internal costing |
| Einerson, Stehlikova (15) | USA $ (2015) | Economic evaluation | 24.9 |  |  | Range: (13-111) | Literature |
| Fernandez, Cronin (18) | USA $ (2012) | Chart review | 60 |  |  |  | Internal costing |
| Frank, Rothschild (21) | USA $ (2012) | Case series | 10.61 |  |  | Hospital charge to patients: 52 | Internal costing |
| Pham, Kim (25) | USA $ (2013) | Economic evaluation | 60 |  | 20 |  | Internal costing |
| Razavi, Carter (62) | USA $ (2013) | Quasi-experimental before-and-after | 7.21 |  | 6.72 |  | Internal costing |
| Reppucci, Meier (27) | USA $ (2021) | Chart review | 632 |  |  |  | Internal costing |
| Rinehart, Lee (28) | USA $ (2014) | Case series | 5.50 |  |  |  | Internal costing |
| Saringcarinkul and Chuasuwan (29) | USA $ (2015) | Case series | 3.59 |  |  | Does not include salaries, infrastructure, training, reagents, and supplies for donor blood collection, storage, and testing. | Internal costing |
| Shafie, Wong (63) | Malaysian Ringgit (2019) | Economic evaluation | 11 |  |  | Federal Government Malaysia. FEES (Medical) (Cost of services) ORDER,  2014 [ | Reference cost |
| Shiru, Abdul (64) | USA $ (2018) | Chart review | 8.40 |  |  |  | Internal costing |
| Stokes, Wordsworth (34) | UK £ (2015) | Microcosting | 8.07 |  |  |  | Internal costing |
| Ural, Volpi-Abadie (65) | USA $ (2016) | Case series | 14 |  |  |  | Internal costing |
| Zhao, Dahlen (44) | USA $ (2018) | Case series | 31 |  |  |  | Reference cost |
| Hall, Pattenden (46) | UK £ (2013) | Case series | 7 |  |  |  | Internal costing |
| Hasan, Khan (47) | USA $ (2018) | Case series | 48.40 |  |  |  | Internal costing |
| Indelen, Kizmaz (66) | USA $ (2018) | Microcosting | 7.21 |  |  |  | Internal costing |
| Kacker, Ness (49) | USA $ (2012( | Economic evaluation |  | 24.77 ^*^ | 14.95^**^ | AHG test,  ^*^Range: (18.58- 30.96)  ^**^Range: (11.21- 18.69) | Reference cost |
| Kleineruschkamp, Meybohm (67) | Euro (2019) | Systematic Review | 11.66 |  |  |  | Literature |
| Kwok, White (52) | UK £ (2013) | Audit |  |  | 3.75 |  | Internal costing |
| Mann, Sim (54) | UK £ (2012) | Case series | 137.22 |  |  | Include cost of transfusion | Internal costing |
| O'Donnell, Shean (56) | USA $ (2018) | Cohort | 126-175 |  |  |  | Internal costing |
| Hildebrand, Binnie (48) | UK £ (2012) | Case series | 30 |  |  |  | Internal costing |

*Legend:* ***^*^****costs are reported in the original currency value and year*

**Table 3: Studies reporting overall unit cost for Groupe and Save (GS) and Crossmatch (CM)**

| **Study** | **Currency** | **Study design** | **Cost per test ^*^(GS&CM)** | **Cost per patient (GS&CM)^*^** | **Notes** | **Costing method** |
| --- | --- | --- | --- | --- | --- | --- |
| Barreto, Singh (8) | USA $ (2017) | Cohort | 5.90 |  |  | Internal costing |
| Barth, Weiss (68) | USA $ (2018) | Case series | 625.39 |  | Range: (± 158.31) | Literature |
| Shafie, Wong (63) | Malaysian Ringgit (2019) | Economic evaluation | 11 |  |  | Not stated |
| Straub, Bauer (69) | Euro (2015) | Economic evaluation |  | 46.75 |  | Literature |
| Tay, Woo (36) | Singapore dollar (2016) | Chart review | 57.10 |  |  | Internal costing |
| Ural, Volpi-Abadie (65) | USA $ (2016) | Case series |  | 36.87 |  |  |
| Volin, Daniel (42) | USA $ (2015) | Economic evaluation | 24.9 |  | Range: (13-111) | Literature |
| Yang, Singhal (70) | Canadian dollars (2015) | Chart review | 40.25 |  | Does not include indirect costs | Internal costing |
| Haleem, Thimmaiah (71) | UK £ (2022) | Case series | 154 |  |  | Not stated |

*Legend:* ***^*^****costs are reported in the original currency and year, GS: Group and Save, CM: Crossmatch*

**Table 4: Studies with economic focus**

| ***Study*** | ***Country*** | ***Aim*** | ***Group and save/Crossmatch as main objective (yes/no)*** | ***Contextual study design*** | ***Type of economic evaluation*** | ***Economic evaluation model*** | ***Main findings*** |
| --- | --- | --- | --- | --- | --- | --- | --- |
| Einerson, Stehlikova (15) | USA | To evaluate the cost-effectiveness of common obstetric transfusion preparedness strategies to prevent emergency-release transfusions. | yes | Economic evaluation | Cost-effectiveness | Decision Tree | In obstetric haemorrhage preparedness, universal type and screen was never cost-effective, with no-testing or selective strategies for high-risk patients offering far lower cost per emergency-release transfusion prevented. |
| Indelen, Kizmaz (66) | Turkey | To analyse the cost of the entire transfusion process in Turkey including evaluation of the cost of transfusion from the perspective of hospital management and determination of savings achieved with the transfusion improvement program | no | Microcosting | NA | NA | The hospital cost of crossmatch was $7.21 and ABO + Rh typing $5.91, forming part of the total $240–$251 per erythrocyte suspension unit. |
| Kacker, Ness (49) | USA | To evaluate the cost-effectiveness of various antigen-matching strategies for chronically transfused sickle cell disease patients | no | Economic evaluation | Cost-effectiveness | Markov | For sickle cell disease, prospective extended antigen matching including ABO/Rh and antibody screen substantially reduced alloimmunisation but at very high incremental cost per event averted. |
| Mafirakureva, Nyoni (58) | Zimbabwe | To assess the unit costs of producing blood in Zimbabwe using an activity-based costing method | no | Macro cost analysis | NA | NA | Blood grouping and crossmatching accounted for US$17.88 (15.1%) of the US$118–$131 cost of producing a unit of blood or red cells. |
| Patel, Edwards (24) | USA | To investigate the cost saving effect of increasing group and save sample storage interval from 7 to 14 days for patients with no history of transfusion or pregnancy in the past 3 months and no history of clinically significant RBC antibodies. | yes | Economic evaluation | Cost benefit | No model | Extending preoperative type and screen sample validity from 7 to 14 days reduced repeat testing and saved an estimated $38,770 annually, with greater savings at longer intervals. |
| Pham, Kim (25) | USA | To analyse the cost-benefit of providing phenotypically matched vs. traditional type and cross every 72 h in patients with placenta accreta and/or placenta previa who are admitted to the hospital prior to scheduled delivery. | yes | Economic evaluation | Cost benefit | Markov | Preparing phenotypically matched RBC units at admission was found to be more cost-beneficial compared to the traditional method of T&C every 72 hours, provided the cost for the matched unit was less than $857.67 USD |
| Shafie, Wong (63) | Malaysia | To estimate the lifetime costs of transfusion-dependent thalassaemia patients in Malaysia from a societal perspective. | no | Economic evaluation | Cost analysis | Markov | In transfusion-dependent thalassaemia, blood group and crossmatch testing cost $2.70 per transfusion, contributing to 13.1% of lifetime healthcare costs. |
| Spillinger, Allen (31) | USA | To evaluate the cost-effectiveness of obtaining preoperative type and screens for common endonasal skull base procedures, and  determine patient and hospital factors associated with receiving blood transfusion | yes | Economic evaluation | Cost-effectiveness | Decision Tree | For endonasal skull base surgery, routine preoperative type and screen was not cost-effective, becoming justified only when transfusion risk exceeded 4.12%. |
| Stokes, Wordsworth (34) | UK | To generate comprehensive estimates of the costs of administering transfusions for the UK National Health Service | no | Microcosting | NA | NA | In two UK hospitals, the average cost of a group and screen was £8.40, with 2.3 tests performed per unit transfused, making test frequency a key driver of laboratory costs. |
| Straub, Bauer (69) | Germany | To analyse the cost-effectiveness Point-of-Care coagulation testing using multiple electrode aggregometry compared to standard laboratory testing in cardiac surgery patient | no | Economic evaluation | Cost-effectiveness | Decision Tree | In cardiac surgery patients, a model including preoperative costs such as blood grouping and crossmatching found that point-of-care coagulation testing reduced transfusions and complications, yielding €288 lower total cost per patient compared with standard laboratory testing. |
| Volin, Daniel (42) | USA | To evaluate the cost-effectiveness of obtaining a preoperative type and screen for common urologic procedures. | yes | Economic evaluation | Cost-effectiveness | Decision Tree | In common urologic procedures, routine preoperative type and screen was not cost-effective unless transfusion risk exceeded 4.12%, with selective or postoperative testing suggested as preferable. |

**Table 5: Detail of surgical specialties**

| Surgical speciality (n=55) | N studies | % |
| --- | --- | --- |
| Orthopaedic surgery | 11 | 20.0 |
| General surgery | 8 | 14.5 |
| Neurosurgery | 4 | 7.3 |
| Obstetrics and gynaecology | 4 | 7.3 |
| Vascular surgery | 4 | 7.3 |
| Cardiothoracic surgery | 3 | 5.5 |
| Anaesthesiology, surgery | 2 | 3.6 |
| Emergency general surgery | 2 | 3.6 |
| Paediatric orthopaedic surgery | 2 | 3.6 |
| Paediatric surgery | 2 | 3.6 |
| Thoracic surgery | 2 | 3.6 |
| Urologic surgery | 2 | 3.6 |
| Bariatric surgery | 1 | 1.8 |
| Breast surgery, oncology | 1 | 1.8 |
| Cardiac and non-cardiac surgery | 1 | 1.8 |
| Colorectal surgery | 1 | 1.8 |
| Gynaecologic oncology surgery | 1 | 1.8 |
| Hepatopancreatobiliary surgery | 1 | 1.8 |
| Neurosurgery, oncology | 1 | 1.8 |
| Oral and maxillofacial surgery | 1 | 1.8 |
| Urologic and surgical oncology | 1 | 1.8 |

**Table 6: Cost results by components (mean)**

|  | N studies | Mean (SD)  (£, 2022) |
| --- | --- | --- |
| Group and save (n=59) | | |
| *Unit cost* | **48** | **58.2 (105.9)** |
| *Cost by component* |  |  |
| Blood typing (ABO) | 6 | 35.1 (30.6) |
| RhD typing | 4 | 17.6 (7.0) |
| Antibody screen | 5 | 36.9 (32.4) |
| Antibody identification | 8 | 90.1 (75.9) |
| *Cost per patient* | 9 | 90.3 (78.1) |
| Crossmatch (n=27) | | |
| Unit cost | **23** | **41.4 (90.9)*** |
| *Cost by component* |  |  |
| Indirect antiglobulin | 3 | 14.8 (9.8) |
| Electronic | 5 | 7.8 (5.6) |
| Group and save + Crossmatch (n=7) | | |
| *Unit cost* | 7 | 106.2 (155.9) |
| *Cost per patient* | 2 | 44.3 (7.6) |

***Legend:*** *SD: standard deviation. ^*^Unit cost by* *Mann et al. includes the cost of transfusion and it was excluded from the mean calculation.*

**Table 7: PRISMA Checklist**

| **Section and Topic** | **Item #** | **Checklist item** | **Location where item is reported (section)** |
| --- | --- | --- | --- |
| **TITLE** | | |  |
| Title | 1 | Identify the report as a systematic review. | title |
| **ABSTRACT** | | |  |
| Abstract | 2 | See the PRISMA 2020 for Abstracts checklist. |  |
| **INTRODUCTION** | | |  |
| Rationale | 3 | Describe the rationale for the review in the context of existing knowledge. | 1 |
| Objectives | 4 | Provide an explicit statement of the objective(s) or question(s) the review addresses. | 1 |
| **METHODS** | | |  |
| Eligibility criteria | 5 | Specify the inclusion and exclusion criteria for the review and how studies were grouped for the syntheses. | 2 |
| Information sources | 6 | Specify all databases, registers, websites, organisations, reference lists and other sources searched or consulted to identify studies. Specify the date when each source was last searched or consulted. | 2 |
| Search strategy | 7 | Present the full search strategies for all databases, registers and websites, including any filters and limits used. | 2 and Supp material A |
| Selection process | 8 | Specify the methods used to decide whether a study met the inclusion criteria of the review, including how many reviewers screened each record and each report retrieved, whether they worked independently, and if applicable, details of automation tools used in the process. | 2 |
| Data collection process | 9 | Specify the methods used to collect data from reports, including how many reviewers collected data from each report, whether they worked independently, any processes for obtaining or confirming data from study investigators, and if applicable, details of automation tools used in the process. | 2 |
| Data items | 10a | List and define all outcomes for which data were sought. Specify whether all results that were compatible with each outcome domain in each study were sought (e.g. for all measures, time points, analyses), and if not, the methods used to decide which results to collect. | 2 and Supp material A |
|  | 10b | List and define all other variables for which data were sought (e.g. participant and intervention characteristics, funding sources). Describe any assumptions made about any missing or unclear information. | 2 and Supp material A |
| Study risk of bias assessment | 11 | Specify the methods used to assess risk of bias in the included studies, including details of the tool(s) used, how many reviewers assessed each study and whether they worked independently, and if applicable, details of automation tools used in the process. | 2 and Supp. material |
| Effect measures | 12 | Specify for each outcome the effect measure(s) (e.g. risk ratio, mean difference) used in the synthesis or presentation of results. | 2 |
| Synthesis methods | 13a | Describe the processes used to decide which studies were eligible for each synthesis (e.g. tabulating the study intervention characteristics and comparing against the planned groups for each synthesis (item #5)). | 2 |
|  | 13b | Describe any methods required to prepare the data for presentation or synthesis, such as handling of missing summary statistics, or data conversions. | 2 |
|  | 13c | Describe any methods used to tabulate or visually display results of individual studies and syntheses. | 2 |
|  | 13d | Describe any methods used to synthesize results and provide a rationale for the choice(s). If meta-analysis was performed, describe the model(s), method(s) to identify the presence and extent of statistical heterogeneity, and software package(s) used. | 2 |
|  | 13e | Describe any methods used to explore possible causes of heterogeneity among study results (e.g. subgroup analysis, meta-regression). | 2 |
|  | 13f | Describe any sensitivity analyses conducted to assess robustness of the synthesized results. | 2 |
| Reporting bias assessment | 14 | Describe any methods used to assess risk of bias due to missing results in a synthesis (arising from reporting biases). | 2 and Suppl material |
| Certainty assessment | 15 | Describe any methods used to assess certainty (or confidence) in the body of evidence for an outcome. | 2 and Supp material B |
| **RESULTS** | | |  |
| Study selection | 16a | Describe the results of the search and selection process, from the number of records identified in the search to the number of studies included in the review, ideally using a flow diagram. | 3 |
|  | 16b | Cite studies that might appear to meet the inclusion criteria, but which were excluded, and explain why they were excluded. | Figure 1 |
| Study characteristics | 17 | Cite each included study and present its characteristics. | 3, tables 1-2 and Supp material B |
| Risk of bias in studies | 18 | Present assessments of risk of bias for each included study. | - |
| Results of individual studies | 19 | For all outcomes, present, for each study: (a) summary statistics for each group (where appropriate) and (b) an effect estimate and its precision (e.g. confidence/credible interval), ideally using structured tables or plots. | Figure 2,3 and Supp material B |
| Results of syntheses | 20a | For each synthesis, briefly summarise the characteristics and risk of bias among contributing studies. | 4 |
|  | 20b | Present results of all statistical syntheses conducted. If meta-analysis was done, present for each the summary estimate and its precision (e.g. confidence/credible interval) and measures of statistical heterogeneity. If comparing groups, describe the direction of the effect. | 3 |
|  | 20c | Present results of all investigations of possible causes of heterogeneity among study results. | 4 |
|  | 20d | Present results of all sensitivity analyses conducted to assess the robustness of the synthesized results. | Figure 1 and 2 Supp material B |
| Reporting biases | 21 | Present assessments of risk of bias due to missing results (arising from reporting biases) for each synthesis assessed. | - |
| Certainty of evidence | 22 | Present assessments of certainty (or confidence) in the body of evidence for each outcome assessed. | 4 and Supp material B |
| **DISCUSSION** | | |  |
| Discussion | 23a | Provide a general interpretation of the results in the context of other evidence. | 4 |
|  | 23b | Discuss any limitations of the evidence included in the review. | 4 |
|  | 23c | Discuss any limitations of the review processes used. | 4 |
|  | 23d | Discuss implications of the results for practice, policy, and future research. | 5 |
| **OTHER INFORMATION** | | |  |
| Registration and protocol | 24a | Provide registration information for the review, including register name and registration number, or state that the review was not registered. | 2 |
|  | 24b | Indicate where the review protocol can be accessed, or state that a protocol was not prepared. | 2 |
|  | 24c | Describe and explain any amendments to information provided at registration or in the protocol. | - |
| Support | 25 | Describe sources of financial or non-financial support for the review, and the role of the funders or sponsors in the review. | Acknowledgement |
| Competing interests | 26 | Declare any competing interests of review authors. | Conflict of interest |
| Availability of data, code and other materials | 27 | Report which of the following are publicly available and where they can be found: template data collection forms; data extracted from included studies; data used for all analyses; analytic code; any other materials used in the review. | - |

*From:*  Page MJ, McKenzie JE, Bossuyt PM, Boutron I, Hoffmann TC, Mulrow CD, et al. The PRISMA 2020 statement: an updated guideline for reporting systematic reviews. BMJ 2021;372:n71. doi: 10.1136/bmj.n71. This work is licensed under CC BY 4.0. To view a copy of this license, visit <https://creativecommons.org/licenses/by/4.0/>

**Table 8: Quality appraisal – Cohort studies**

| **Questions from CASP (cohort study checklist) *(72)*** | **Did the study address a clearly focused issue?** | **Was the cohort recruited in an acceptable way?** | **Was the exposure accurately measured to minimise bias?** | **Was the outcome accurately measured to minimise bias?** | **Have the authors identified all important confounding factors?** | **Have they taken account of the confounding factors in the design and/or analysis?** | **Was the follow up of subjects complete enough?** | **Was the follow up of subjects long enough?** |
| --- | --- | --- | --- | --- | --- | --- | --- | --- |
| Kirschen, Dayton (50) | Yes | Yes | Yes | Yes | Yes | Yes | Yes | Yes |
| Kwok, White (52) | Yes | Yes | Yes | Yes | Can't tell | No | Yes | Yes |
| Machado, Loureiro (53) | Yes | Yes | Yes | Yes | Yes | Yes | Yes | Yes |
| McKenna and Abdelaal (55) | Yes | Yes | Yes | Yes | Yes | Yes | Yes | Yes |
| O'Donnell, Shean (56) | Yes | Yes | Yes | Yes | Yes | Yes | Yes | Yes |
| Steele, Herman (33) | Yes | Yes | Yes | Yes | Yes | Can't tell | Yes | Yes |
| Stokes, Wordsworth (34) | Yes | Yes | Yes | Yes | Yes | Yes | Yes | Yes |
| Tay, Woo (36) | Yes | Yes | Yes | Yes | Yes | Yes | Yes | Yes |
| Thomson, Ross (37) | Yes | Yes | Yes | Yes | Yes | No | Yes | Yes |
| Finley, Fay (19) | Yes | Yes | Yes | Yes | Can't tell | Can't tell | Yes | Yes |
| Strockbine, Gehrie (35) | Yes | Yes | Yes | Yes | Can't tell | Can't tell | Yes | Yes |
| Razavi, Carter (62) | Yes | Yes | Yes | Yes | Can't tell | Can't tell | Yes | Yes |
| Indelen, Kizmaz (66) | Yes | Yes | Yes | Yes | No | No | Yes | Yes |
| Wilson, Young (43) | Yes | Yes | Yes | Yes | Can't tell | No | Yes | Yes |
| Shiru, Abdul (64) | Yes | Yes | Yes | Yes | No | No | Yes | Yes |
| Stangenberg, Curran (32) | Yes | Yes | Yes | Yes | Yes | Yes | Yes | Yes |
| Tjaden, Codispoti (38) | Yes | Yes | Yes | Yes | Can't tell | Can't tell | Yes | Yes |
| Vestermark, Rowe (41) | Yes | Yes | Yes | Yes | Yes | Yes | Yes | Yes |
| Tunthanathip, Sae-Heng (39) | Yes | Yes | Yes | Yes | Can't tell | Can't tell | Yes | Yes |
| Barreto, Singh (8) | Yes | Yes | Yes | Yes | Can't tell | No | Can't tell | Yes |
| Chu, Wagholikar (13) | Yes | Yes | Yes | Yes | Can't tell | No | Yes | Yes |
| Obaidallah, Downie (59) | Yes | Yes | Yes | Yes | Can't tell | No | Can't tell | Yes |
| Garg, Coleman (22) | Yes | Yes | Yes | Yes | Can't tell | No | Yes | Yes |
| Reppucci, Meier (27) | Yes | Yes | Yes | Yes | Yes | Yes | Yes | Yes |
| Bawazir and Dakkam (11) | Yes | Yes | Yes | Can't tell | Can't tell | No | Can't tell | Yes |
| Yang, Singhal (70) | Yes | Yes | Yes | Yes | Can't tell | No | Can't tell | Can't tell |
| Kretzmer, Damola (51) | Yes | Yes | Yes | Yes | Can't tell | No | Can't tell | Can't tell |
| Azizgolshani, Porter (5) | Yes | Yes | Yes | Yes | Yes | Yes | Yes | Yes |
| Fernandez, Cronin (18) | Yes | Yes | Can't tell | Can't tell | Can't tell | No | Can't tell | Can't tell |
| Bamford, Hall (7) | Yes | Yes | Yes | Yes | No | No | Yes | Yes |
| Baig, Sarma (6) | Yes | Yes | Yes | Yes | No | No | Yes | Yes |
| Chung, Hur (60) | Yes | Yes | Yes | Yes | No | No | Yes | Yes |
| Cushing, DeSimone (61) | Yes | Yes | Yes | Yes | No | No | Yes | Yes |
| Mafirakureva, Nyoni (58) | Yes | Yes | Yes | Yes | No | Can't tell | Yes | Yes |

**Table 9: Quality appraisal – Systematic reviews studies**

| **Questions from CASP (systematic review checklist – section A) *(72)*** | **Are the results of the review valid?** | **Did the authors look for the right type of papers?** | **Do you think all the important, relevant studies were included?** | **Did the review’s authors do enough to assess quality of the included studies?** |
| --- | --- | --- | --- | --- |
| Fadel, Patel (16) | Yes | Yes | Can't tell | No |
| Kleineruschkamp, Meybohm (67) | Yes | Yes | Yes | Can't tell |

**Table 10: Quality appraisal – Economic Evaluation studies**

| Questions from Drummond’s checklist (73) | Was a well-defined question posed in an answerable form? | Was a comprehensive description of the competing alternatives given (i.e. who did what to whom, where, and how often)? | Was the effectiveness of the programme or services established? | Were all the important and relevant costs and consequences for each alternative identified? | Were costs and consequences measured accurately in appropriate physical units (e.g. hours of nursing time, etc)? | Were the cost and consequences valued credibly? | Were costs and consequences adjusted for differential timing? | Was an incremental analysis of costs and consequences of alternatives performed? | Was allowance made for uncertainty in the estimates of costs and consequences? | Did the presentation and discussion of study results include all issues of concern to users? |
| --- | --- | --- | --- | --- | --- | --- | --- | --- | --- | --- |
| Pham, Kim (25) | Yes | Yes | Yes | Yes | Yes | Yes | No | Yes | Yes | Yes |
| Shafie, Wong (63) | Yes | No | No | Yes | Yes | Yes | No | No | Yes | Yes |
| Spillinger, Allen (31) | Yes | Yes | Yes | Yes | Yes | Yes | No | Yes | Yes | Yes |
| Volin, Daniel (42) | Yes | Yes | Yes | Yes | Yes | Yes | No | Yes | Yes | Yes |
| Kacker, Ness (49) | Yes | Yes | Yes | Yes | Yes | Yes | Yes | Yes | Yes | Yes |
| Patel, Edwards (24) | Yes | Yes | Yes | Yes | Yes | Yes | No | Yes | Yes | Yes |
| Straub, Bauer (69) | Yes | Yes | Yes | Yes | Yes | Yes | No | Yes | Yes | Yes |
| Einerson, Stehlikova (15) | Yes | Yes | Yes | Yes | Can't tell | Yes | No | Yes | Yes | Yes |

**Table 11: Quality appraisal – Case series studies**

| **Questions from JBI case series Checklist (74)** | **1. Clear criteria for inclusion?** | **2. Condition measured in a standard, reliable way?** | **3. Valid methods used for identification of the condition?** | **4. Consecutive inclusion of participants?** | **5. Complete inclusion of participants?** | **6. Clear reporting of demographics?** | **7. Clear reporting of clinical information?** | **8. Outcomes or follow up results clearly reported?** | **9. Clear reporting of presenting site(s)/clinic(s) demographic information?** | **10. Statistical analysis appropriate?** |
| --- | --- | --- | --- | --- | --- | --- | --- | --- | --- | --- |
| Adams, Baldwin (1) | Yes | Yes | Yes | Yes | Yes | Yes | Yes | Yes | Yes | Yes |
| Adams, Cahill (2) | Yes | Yes | Yes | Yes | Yes | Yes | Yes | Yes | Yes | Yes |
| Al-Musawi, Reece (3) | Yes | Yes | Yes | Yes | Yes | Unclear | Yes | Yes | Yes | Unclear |
| Alyacoubi, Taj (4) | Yes | Yes | Yes | Yes | Yes | Yes | Yes | Yes | Yes | Yes |
| Barrett-Lee, Vatish (9) | Yes | Yes | Yes | Yes | Yes | Unclear | Yes | Yes | Yes | Unclear |
| Barth, Weiss (68) | Yes | Yes | Yes | Yes | Yes | Unclear | Yes | Yes | Yes | Yes |
| Batt, Chambers (10) | Yes | Yes | Yes | Unclear | Unclear | Unclear | Yes | Yes | Yes | Unclear |
| Christopher, Verhey (12) | Yes | Yes | Yes | Yes | Yes | Unclear | Yes | Yes | Yes | Yes |
| Compton, Szklarski (14) | Yes | Yes | Yes | Yes | Yes | N/A | Yes | Yes | Yes | Yes |
| Farrell, Hall (17) | Yes | Yes | Yes | Yes | Yes | Yes | Yes | Yes | Yes | Unclear |
| Fong, Rodriguez (20) | Yes | Yes | Yes | Yes | Yes | Unclear | Yes | Yes | Yes | Unclear |
| Frank, Rothschild (21) | Yes | Yes | Yes | Yes | Yes | N/A | Yes | Yes | Yes | Yes |
| Hainsworth, Tracy (45) | Yes | Yes | Yes | Yes | Yes | Unclear | Yes | Yes | Yes | Unclear |
| Haleem, Thimmaiah (71) | Yes | Yes | Yes | Yes | Yes | Yes | Yes | Yes | Yes | Yes |
| Hall, Pattenden (46) | Yes | Yes | Yes | Yes | Yes | N/A | Yes | Yes | Yes | Yes |
| Hasan, Khan (47) | Yes | Yes | Yes | Yes | No | Yes | Yes | Yes | Yes | Yes |
| Hildebrand, Binnie (48) | Yes | Yes | Yes | Yes | Yes | Yes | Yes | Yes | Yes | Unclear |
| Mann, Sim (54) | Yes | Yes | Yes | Yes | Yes | N/A | Yes | Yes | Yes | Unclear |
| Mazonson, Efrusy (57) | Yes | Yes | Yes | Yes | Yes | Yes | Yes | Yes | Yes | Yes |
| Parker, Mahawar (23) | Yes | Yes | Yes | Yes | Yes | N/A | Yes | Yes | Yes | Unclear |
| Rayborn, Turner (26) | Unclear | Yes | Yes | Unclear | Unclear | Yes | Yes | Yes | Yes | Unclear |
| Rinehart, Lee (28) | Yes | Yes | Yes | Yes | Yes | N/A | Yes | Yes | Yes | Yes |

**Figure 1: Subgroup analysis of GS test, by country and valuation type**

**
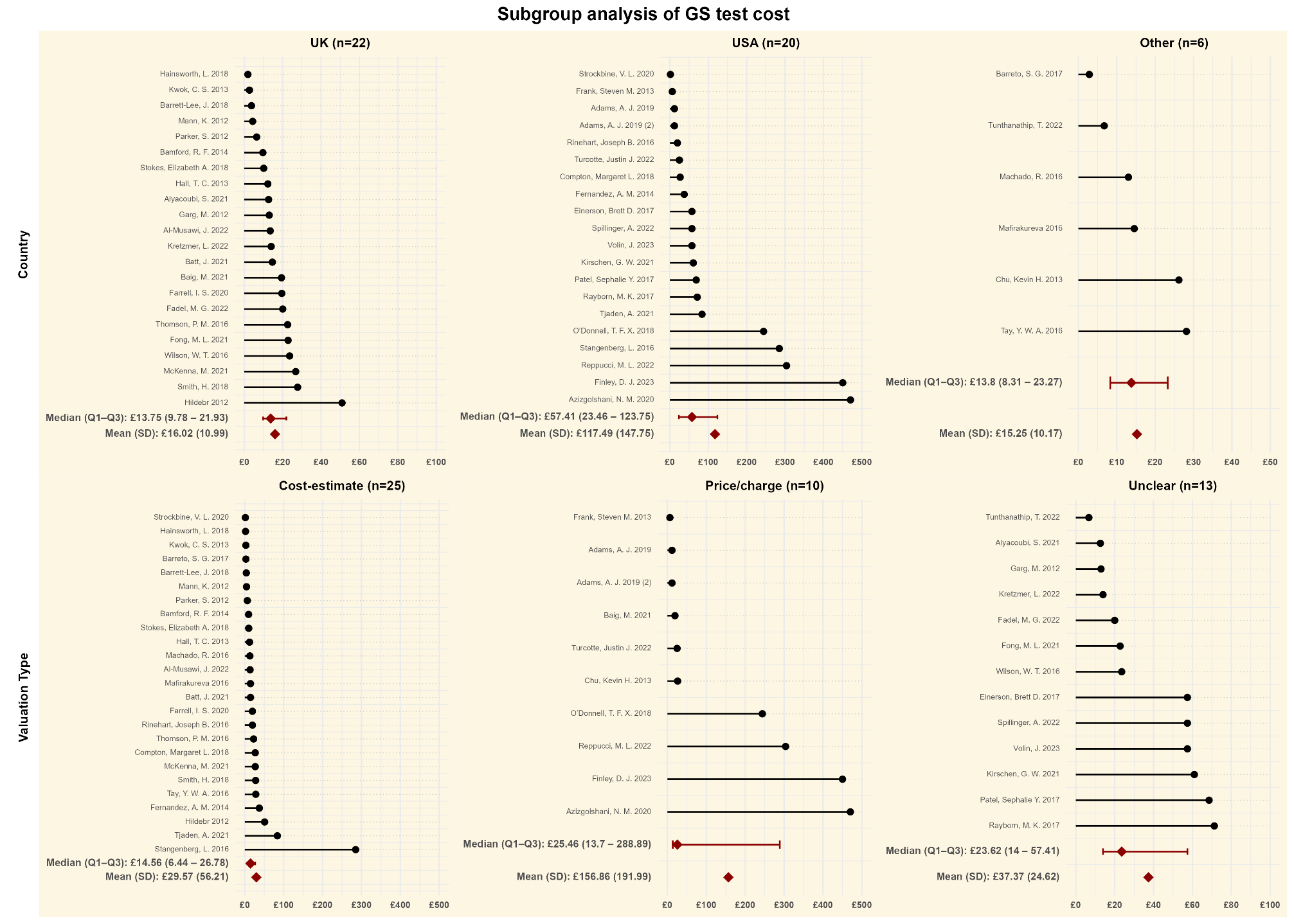
**

**Figure 2: Subgroup analysis of CM test, by country and valuation type**

**
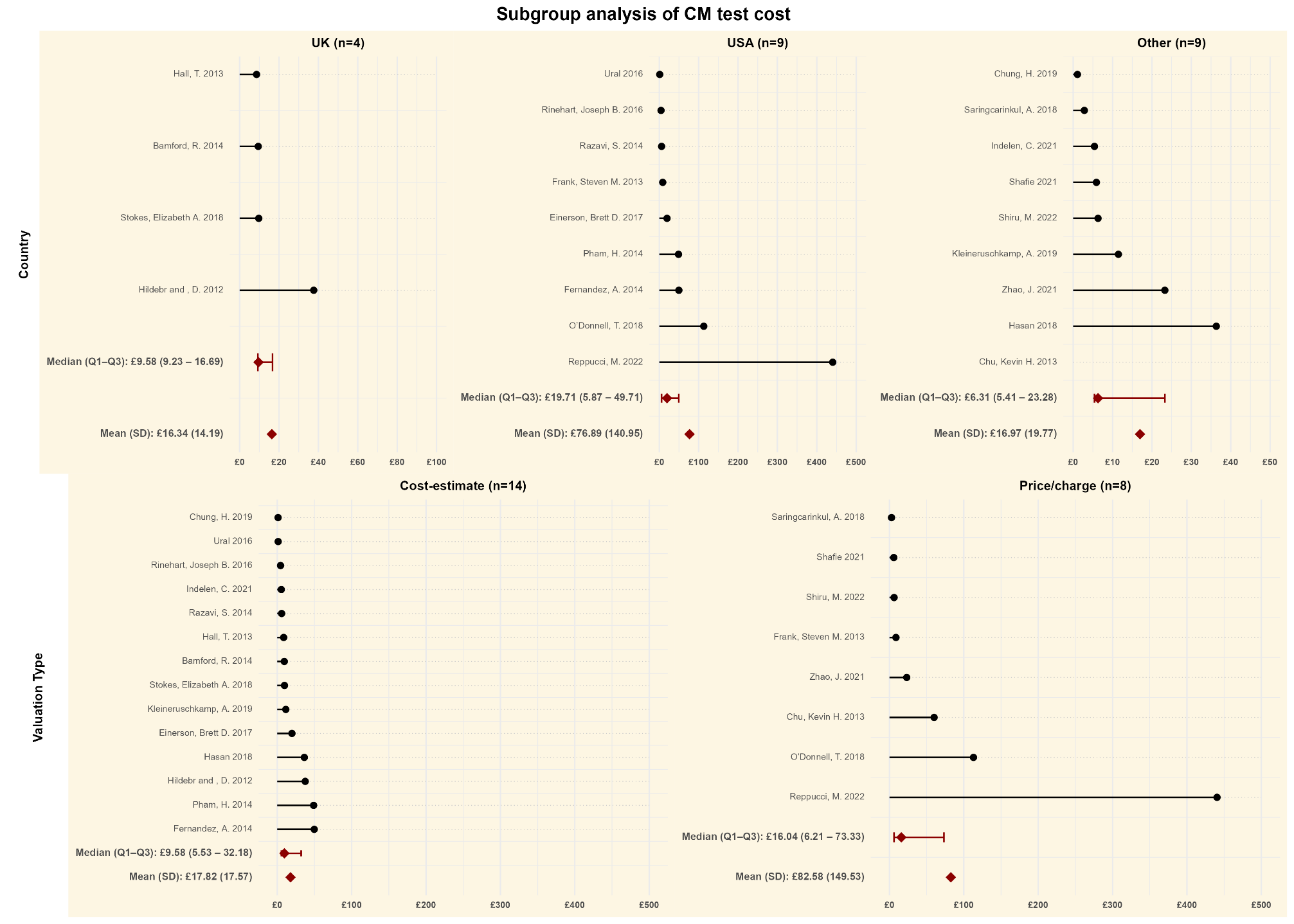
**

**References:**

1. Adams AJ, Baldwin KD, Arkader A, Sankar WN. Assessing the Need for Common Perioperative Laboratory Tests in Pediatric Patients With Femoral Shaft Fractures. Journal of Pediatric Orthopaedics. 2019;39(6):E456-E61.

2. Adams AJ, Cahill PJ, Flynn JM, Sankar WN. Utility of Perioperative Laboratory Tests in Pediatric Patients Undergoing Spinal Fusion for Scoliosis. Spine Deformity. 2019;7(6):875-82.

3. Al-Musawi J, Reece I, Chen JY, Britton C, Shakweh E, Vutipongsatorn K, et al. Perioperative group and save testing are not routinely indicated for emergency laparoscopic appendicectomy and laparoscopic hernia repairs: A North West London retrospective study. Journal of Perioperative Practice. 2022:17504589221110333.

4. Alyacoubi S, Taj T, Raza I. Routine group and save screening prior to emergency laparoscopic surgery. Annals of the Royal College of Surgeons of England. 2021;103(6):412-4.

5. Azizgolshani NM, Porter ED, Fay KA, Dunbar NM, Hasson RM, Millington TM, et al. Preoperative Type and Screen is Unnecessary in Elective Anatomic Lung Resection and Esophagectomy. Journal of Surgical Research. 2020;255:411-9.

6. Baig M, Sarma D, Ng V, Shortland T, Sood S. Health Economics and Safety of Frontline Carers in the COVID-19 Era: Time to Abolish Routine Group and Save For Emergency Appendicectomies? BRITISH JOURNAL OF SURGERY. 2021;108.

7. Bamford RF, Hall A, Loftus IM, Thompson MM, Black SA. Rationalising cross-match requests in vascular surgery is safe and cost effective. Journal of perioperative practice. 2014;24(9):206-9.

8. Barreto SG, Singh A, Perwaiz A, Singh T, Singh MK, Chaudhary A. Maximum surgical blood order schedule for pancreatoduodenectomy: A long way from uniform applicability&excl. Future Oncology. 2017;13(9):799-807.

9. Barrett-Lee J, Vatish J, Vazirian-Zadeh M, Waterland P. Routine blood group and antibody screening prior to emergency laparoscopy. ANNALS OF THE ROYAL COLLEGE OF SURGEONS OF ENGLAND. 2018;100(4):322-5.

10. Batt J, Chambers A, Mason J, Mullan M. Is group and save still a necessary test in the preoperative workup for breast cancer surgery? Journal of perioperative practice. 2021;31(5):187-90.

11. Bawazir WM, Dakkam FM. Enhancing the utilization of packed red blood cells stock in maternity hospitals. Saudi Medical Journal. 2020;41(6):628-34.

12. Christopher ZK, Verhey JT, Bruce MR, Bingham JS, Spangehl MJ, Clarke HD, et al. Routine Type and Screens Are Unnecessary in Primary Total Joint Arthroplasty: Follow-up After a Change in Practice. Arthroplasty Today. 2023;19 (no pagination).

13. Chu KH, Wagholikar AS, Greenslade JH, O'Dwyer JA, Brown AF. Sustained reductions in emergency department laboratory test orders: impact of a simple intervention. Postgraduate Medical Journal. 2013;89(1056):566-71.

14. Compton ML, Szklarski PC, Booth GS. Duplicate Type and Screen Testing: Waste in the Clinical Laboratory. Archives of Pathology & Laboratory Medicine. 2018;142(3):358-63.

15. Einerson BD, Stehlikova Z, Nelson RE, Bellows BK, Kensaku K, Clark EAS, et al. Transfusion Preparedness Strategies for Obstetric Hemorrhage: A Cost-Effectiveness Analysis. Obstetrics & Gynecology. 2017;130(6):1347-55.

16. Fadel MG, Patel I, O'Leary L, Behar N, Brewer J. Requirement of preoperative blood typing for cholecystectomy and appendectomy: a systematic review. Langenbeck's Archives of Surgery. 2022;407(6):2205-16.

17. Farrell IS, Hall J, Hill J. Cost analysis of blood group and antibody screening for emergency appendicectomy: Should we stop? World Journal of Laparoscopic Surgery. 2020;13(3):128-9.

18. Fernandez AM, Cronin J, Greenberg RS, Heitmiller ES. Pediatric preoperative blood ordering: When is a type and screen or crossmatch really needed? Paediatric Anaesthesia. 2014;24(2):146-50.

19. Finley DJ, Fay KA, Porter ED, Hasson RM, Millington TM, Phillips JD. Reducing Unnecessary Type and Screens Prior to Thoracic Surgery: A Quality Improvement Initiative. Journal of Surgical Research. 2023;283:743-50.

20. Fong ML, Rodriguez DU, Elberm H, Berry DP. Are Type and Screen Samples Routinely Necessary Before Laparoscopic Cholecystectomy? JOURNAL OF GASTROINTESTINAL SURGERY. 2021;25(2):447-51.

21. Frank SM, Rothschild JA, Masear CG, Rivers RJ, Merritt WT, Savage WJ, et al. Optimizing preoperative blood ordering with data acquired from an anesthesia information management system. Anesthesiology. 2013;118(6):1286-97.

22. Garg M, Coleman M, Dhariwal DK. Are blood investigations, or group and save, required before orthognathic surgery? British Journal of Oral and Maxillofacial Surgery. 2012;50(7):611-3.

23. Parker S, Mahawar K, Balupuri S, Boyle M, Small P. Routine group and save unnecessary for gastric band: Surgery: A retrospective case review audit of 1018: Bariatric patients. Clinical Obesity. 2012;2(3-4):73-7.

24. Patel SY, Edwards DA, Boulware DC, Serdiuk A, Cook SJ, Benson K, et al. A novel approach to improving efficiency and cost saving in preoperative blood preparation. Transfusion. 2017;57(12):3035-9.

25. Pham HP, Kim CH, Schwartz J. Phenotypically matched vs. traditional screen method for preparing red blood cell units in patients with abnormal placentation: a decision analysis approach. Vox sanguinis. 2014;107(4):399-406.

26. Rayborn MK, Turner JL, Park SG. Cost effectiveness of preoperative screening for healthy patients undergoing robotic hysterectomy. Journal of perioperative practice. 2017;27(6):129-34.

27. Reppucci ML, Meier M, Stevens J, Shirek G, Kulungowski AM, Acker SN. Incidence of and risk factors for perioperative blood transfusion in infants undergoing index pediatric surgery procedures. Journal of Pediatric Surgery. 2022;57(6):1067-71.

28. Rinehart JB, Lee TC, Kaneshiro K, Tran MH, Sun C, Kain ZN. Perioperative blood ordering optimization process using information from an anesthesia information management system. Transfusion. 2016;56(4):938-45.

29. Saringcarinkul A, Chuasuwan S. Maximum Surgical Blood Order Schedule for Elective Neurosurgery in a University Teaching Hospital in Northern Thailand. Asian J Neurosurg. 2018;13(2):329-35.

30. Smith H, Falconer R, Szczachor J, Ahmad S. Routine preoperative group and save for TURP and TURBT - need and cost effectiveness. Journal of Clinical Urology. 2018;11(1):33-7.

31. Spillinger A, Allen M, Karabon P, Hojjat H, Shenouda K, Hussein IH, et al. Cost-Effectiveness of Routine Type and Screens in Select Endonasal Skull Base Surgeries. Journal of Neurological Surgery, Part B: Skull Base. 2022;83(Supplement 2):E449-E58.

32. Stangenberg L, Curran T, Shuja F, Rosenberg R, Mahmood F, Schermerhorn M. Development of a risk prediction model for transfusion in carotid endarterectomy and demonstration of cost-saving potential by avoidance of "type and screen". BRITISH JOURNAL OF SURGERY. 2016;103:23-4.

33. Steele J, Herman M, Norfolk A, Haspel RL. Clinical consequences of a 30-day interval between antibody identifications. Transfusion. 2023;63(1):30-4.

34. Stokes EA, Wordsworth S, Staves J, Mundy N, Skelly J, Radford K, et al. Accurate costs of blood transfusion: a microcosting of administering blood products in the United Kingdom National Health Service. Transfusion. 2018;58(4):846-53.

35. Strockbine VL, Gehrie EA, Qiuping Z, Guzzetta CE. Reducing Unnecessary Phlebotomy Testing Using a Clinical Decision Support System. Journal for Healthcare Quality: Promoting Excellence in Healthcare. 2020;42(2):98-105.

36. Tay YWA, Woo YL, Tan HCA. Routine pre-operative group cross-matching in total knee arthroplasty: A review of this practice in an Asian population. KNEE. 2016;23(2):306-9.

37. Thomson PM, Ross J, Mukherjee S, Mohammadi B. Are Routine Blood Group and Save Samples Needed for Laparoscopic Day Case Surgery? World journal of surgery. 2016;40(6):1295-8.

38. Tjaden A, Codispoti N, Yang LC, Pham T. Examining the Utility and Cost of Routine Type and Screen Prior to Minimally Invasive Hysterectomy. JSLS : Journal of the Society of Laparoendoscopic Surgeons. 2021;25(3).

39. Tunthanathip T, Sae-Heng S, Oearsakul T, Kaewborisutsakul A, Taweesomboonyat C. Economic impact of a machine learning-based strategy for preparation of blood products in brain tumor surgery. PLoS ONE. 2022;17(7 July) (no pagination).

40. Turcotte JJ, Holbert SE, Orlov MD, Patton CM. Development and validation of a risk-based algorithm for preoperative type and screen testing in spine surgery. Spine Journal. 2022;22(9):1472-80.

41. Vestermark GL, Rowe TM, Martin JR, Odum SM, Springer BD, Fehring TK. In the Era of Tranexamic Acid, are Type and Screens for Primary Total Joint Arthroplasty Obsolete? Journal of Arthroplasty. 2020;35(9):2363-6.

42. Volin J, Daniel J, Walter B, Herndon P, Tran D, Blumline J, et al. Cost-effectiveness of routine type and screens in select urological surgeries. International Urology and Nephrology. 2023.

43. Wilson WT, Young AM, Fivey P. Minimising costs in spinal surgery: is group & save testing justified in lumbar decompression surgery? J. 2016;2(4):277-80.

44. Zhao J, Dahlen T, Edgren G. Costs associated with transfusion therapy in patients with myelodysplastic syndromes in Sweden: a nationwide retrospective cohort study. Vox Sanguinis. 2021;116(5):581-90.

45. Hainsworth L, Tracy J, Spolton-Dean C, Donaldson O. Are on-the-day group and save samples required for elective shoulder, hip and knee arthroplasties? Annals of the Royal College of Surgeons of England. 2018:1-3.

46. Hall TC, Pattenden C, Hollobone C, Pollard C, Dennison AR. Blood transfusion policies in elective general surgery: How to optimise cross-match-to-transfusion ratios. Transfusion Medicine and Hemotherapy. 2013;40(1):27-31.

47. Hasan O, Khan EK, Ali M, Sheikh S, Fatima A, Rashid HU. "It's a precious gift, not to waste": is routine cross matching necessary in orthopedics surgery? Retrospective study of 699 patients in 9 different procedures. BMC Health Services Research. 2018;18(1):1-7.

48. Hildebrand DR, Binnie NR, Aly EH. Is routine blood cross-matching necessary in elective laparoscopic colorectal surgery? International Journal Of Surgery. 2012;10(2):92-5.

49. Kacker S, Ness PM, Savage WJ, Frick KD, Shirey RS, King KE, et al. Cost-effectiveness of prospective red blood cell antigen matching to prevent alloimmunization among sickle cell patients. Transfusion. 2014;54(1):86-97.

50. Kirschen GW, Dayton SM, Blakey-Cheung S, Pearl ML. Which patients on a gynecologic oncology service will require perioperative transfusion? A single-center retrospective cohort study. Clinical and Experimental Obstetrics and Gynecology. 2021;48(1):47-52.

51. Kretzmer L, Damola A, Libotte C, Ehsanullah SA, Jones A, Apakama I. Is Group and Saving before the Modern Bipolar Transurethral Resection of the Prostate Still Necessary? Journal of Endoluminal Endourology. 2022;5(2):e44-e8.

52. Kwok CS, White SH, Brammar TJ. Group and save is safe and cost effective in elective total hip arthroplasty - completion of an audit cycle. Transfusion Medicine. 2013;23(1):61-3.

53. Machado R, Loureiro L, Antunes I, Coutinho J, Almeida R. Endovascular Treatment of Aortic Aneurysms and Blood Transfusion. What do We Need? Acta Medica Portuguesa. 2016;29(5):310-4.

54. Mann K, Sim I, Ali T, Chong P, Leopold P, Hatrick A, et al. Removing the need for crossmatched blood in elective EVAR. European Journal of Vascular and Endovascular Surgery. 2012;43(3):282-5.

55. McKenna M, Abdelaal A. Group & save sampling in lumbar decompression: A review into current practice. Journal of perioperative practice. 2021;31(1-2):15-7.

56. O'Donnell TFX, Shean KE, Deery SE, Bodewes TCF, Wyers MC, O'Brien KL, et al. A preoperative risk score for transfusion in infrarenal endovascular aneurysm repair to avoid type and cross. Journal of Vascular Surgery. 2018;67(2):442-8.

57. Mazonson P, Efrusy M, Santas C, Ziman A, Burner J, Roseff S, et al. The HI-STAR study: resource utilization and costs associated with serologic testing for antibody-positive patients at four United States medical centers. Transfusion. 2014;54(2):271-7.

58. Mafirakureva N, Nyoni H, Nkomo SZ, Jacob JS, Chikwereti R, Musekiwa Z, et al. The costs of producing a unit of blood in Zimbabwe. Transfusion. 2016;56(3):628-36.

59. Obaidallah N, Downie H, Colavecchia C, Callum J, Lin Y. Implementation of a blood bank generated tube for second blood group determination: Challenges, yield, and cost. Transfusion. 2022;62(4):784-90.

60. Chung HJ, Hur M, Choi SG, Lee HK, Lee S, Kim H, et al. Benefits of VISION Max automated cross-matching in comparison with manual cross-matching: A multidimensional analysis. PLoS ONE [Electronic Resource]. 2019;14(12):e0226477.

61. Cushing MM, DeSimone RA, Goel R, Hsu YMS, Parra P, Racine‐Brzostek SE, et al. The impact of Daratumumab on transfusion service costs. Transfusion. 2019;59(4):1252-8.

62. Razavi SA, Carter AB, Puskas JD, Gregg SR, Aziz IF, Buchman TG. Reduced red blood cell transfusion in cardiothoracic surgery after implementation of a novel clinical decision support tool. Journal of the American College of Surgeons. 2014;219(5):1028-36.

63. Shafie AA, Wong JHY, Ibrahim HM, Mohammed NS, Chhabra IK. Economic burden in the management of transfusion-dependent thalassaemia patients in Malaysia from a societal perspective. Orphanet Journal of Rare Diseases. 2021;16(1):1-12.

64. Shiru MM, Abdul IF, Ubom AE, Olabinjo AO, Oriji PC, Fiebai PO. Blood reservation and utilisation practice for Caesarean section in Ilorin, Nigeria. Tropical Doctor. 2022.

65. Ural KG, Volpi-Abadie J, Owen G, Gilly G, Egger AL, Scuderi-Porter H. Tailoring the Blood Ordering Process for Cardiac Surgical Cases Using an Institution-Specific Version of the Maximum Surgical Blood Order Schedule. Seminars in Cardiothoracic & Vascular Anesthesia. 2016;20(1):93-9.

66. Indelen C, Kizmaz YU, Kar A, Shander A, Kirali K. The cost of one unit blood transfusion components and cost-effectiveness analysis results of transfusion improvement program. TURK GOGUS KALP DAMAR CERRAHISI DERGISI-TURKISH JOURNAL OF THORACIC AND CARDIOVASCULAR SURGERY. 2021;29(2):150-7.

67. Kleineruschkamp A, Meybohm P, Straub N, Zacharowski K, Choorapoikayil S. A model-based cost-effectiveness analysis of Patient Blood Management. Blood Transfusion. 2019;17(1):16-26.

68. Barth M, Weiss C, Schmieder K. Red blood cell transfusion probability and associated costs in neurosurgical procedures. Acta Neurochirurgica. 2018;160:1483-9.

69. Straub N, Bauer E, Agarwal S, Meybohm P, Zacharowski K, Hanke AA, et al. Cost-effectiveness of POC coagulation testing using multiple electrode aggregometry. Clinical Laboratory. 2016;62(6):1167-78.

70. Yang MMH, Singhal A, Au N, Hengel AR. Impact of preoperative laboratory investigation and blood cross-match on clinical management of pediatric neurosurgical patients. CHILDS NERVOUS SYSTEM. 2015;31(4):533-9.

71. Haleem S, Thimmaiah R, Nagrath N, Gowda D, Bhimarasetty C, Mehta JS. The impact of blood conservation techniques on transfusion requirements for posterior adolescent idiopathic scoliosis corrections: do we need a routine cross-match for the operation? Spine Deformity. 2022;10(3):589-93.

72. CASP. Critical Appraisal Skills Programme (2023). 2023.

73. Drummond M, Sculpher M, Torrance G, Stoddart G. Drummond’s check-list for assessing economic evaluations. Methods for the Economic Evaluation of Healthcare Programs. 2005.

74. Moola S, Munn Z, Tufanaru C, Aromataris E, Sears K, Sfetcu R, et al. Systematic reviews of etiology and risk. JBI manual for evidence synthesis. 2020;1:217-69.
